# Marine DNA methylation patterns are associated with microbial community composition and inform virus-host dynamics

**DOI:** 10.1186/s40168-022-01340-w

**Published:** 2022-09-28

**Authors:** Hoon Je Seong, Simon Roux, Chung Yeon Hwang, Woo Jun Sul

**Affiliations:** 1 Department of Systems Biotechnology, Chung-Ang University, Anseong, Republic of Korea; 2 DOE Joint Genome Institute, Lawrence Berkeley National Laboratory, Berkeley, CA USA; 3 School of Earth and Environmental Sciences and Research Institute of Oceanography, Seoul National University, Seoul, Republic of Korea

**Keywords:** Ocean microbiome, DNA methylation, Metagenome-assembled genome, Host–phage, Long-read sequencing

## Abstract

**Background:**

DNA methylation in prokaryotes is involved in many different cellular processes including cell cycle regulation and defense against viruses. To date, most prokaryotic methylation systems have been studied in culturable microorganisms, resulting in a limited understanding of DNA methylation from a microbial ecology perspective. Here, we analyze the distribution patterns of several microbial epigenetics marks in the ocean microbiome through genome-centric metagenomics across all domains of life.

**Results:**

We reconstructed 15,056 viral, 252 prokaryotic, 56 giant viral, and 6 eukaryotic metagenome-assembled genomes from northwest Pacific Ocean seawater samples using short- and long-read sequencing approaches. These metagenome-derived genomes mostly represented novel taxa, and recruited a majority of reads. Thanks to single-molecule real-time (SMRT) sequencing technology, base modification could also be detected for these genomes. This showed that DNA methylation can readily be detected across dominant oceanic bacterial, archaeal, and viral populations, and microbial epigenetic changes correlate with population differentiation. Furthermore, our genome-wide epigenetic analysis of *Pelagibacter* suggests that GANTC, a DNA methyltransferase target motif, is related to the cell cycle and is affected by environmental conditions. Yet, the presence of this motif also partitions the phylogeny of the *Pelagibacter* phages, possibly hinting at a competitive co-evolutionary history and multiple effects of a single methylation mark.

**Conclusions:**

Overall, this study elucidates that DNA methylation patterns are associated with ecological changes and virus-host dynamics in the ocean microbiome.

Video Abstract

**Supplementary Information:**

The online version contains supplementary material available at 10.1186/s40168-022-01340-w.

## Background

DNA methylation is an important epigenetic modification that is involved in prokaryotic processes such as gene expression regulation, virulence, DNA mismatch repair, and cell-cycle regulation [1, 2]. While there has been increasing interest in the role of prokaryotic methylation systems in bacterial genetics, physiology, and ecology, the function and implications of prokaryotic methylation systems in environmental conditions are poorly understood. This is primarily because previous research has focused extensively on culturable prokaryotes, whereas the majority of environmental bacteria are currently not culturable under laboratory conditions.

In prokaryotes, DNA methylation has been largely associated with restriction-modification (RM) systems, which protect host cells against invasion by viruses or against horizontal gene transfer of extracellular DNA by distinguishing host DNA from sequence-specific DNA methylation [3]. Furthermore, DNA methyltransferase (MTases) that methylate nucleotides without cognate restriction enzymes (REases) are referred to as orphan MTases. Some well-studied orphan MTases play important physiological roles beyond RM systems, including transcriptional regulation and cell phenotype variations [1, 2, 4, 5].

The advent of long-read sequencing technology has opened a new era in methylation research and has enabled the identification of chemical modifications in the DNA structure. Nanopore sequencing enables the identification of all known base modifications [*N*^6^-methyladenosine (m^6^A), *N*^4^-methylcytosine (m^4^C), and *N*^5^-methylcytosine (m^5^C)], whereas the ability of single-molecule real-time (SMRT) sequencing to identify these modifications remains currently limited [6]. Conversely, SMRT sequencing can identify DNA methylation at a single-nucleotide resolution, whereas nanopore sequencing has limitations in detecting accurate modification signals due to current level noise [6]. In the metagenomic perspectives, this methylation information has been used in the past to improve prokaryotic genome binning [7, 8]. However, outside these few examples, few attempts have been made to apply long-read metagenomic sequencing to detect DNA methylation directly in the environmental microbiome [9], and link methylation patterns to ongoing eco-evolutionary processes.

Here, we applied meta-epigenomic analysis to genome-centric metagenomics of the open ocean microbial communities in the northwest Pacific Ocean to reveal the role of DNA methylation in environmental microbial communities. Specifically, we explored how molecular mechanisms for base methylation changed in frequency and patterns across taxa and genomes, both across entire communities and within individual populations, and investigated whether these changes could be associated with cell regulation mechanisms and/or inter-organismal interactions. We report that the DNA methylome is differentiated by taxonomic lineage and is affected by the complexity of the community, i.e., the co-existence of multiple closely related strains. We further link methylation patterns to cell cycle regulation and phage defense for *Pelagibacter* genomes, highlighting the multiple roles played by DNA methylation in one of the dominant bacteria of the marine environment.

## Results

### Novel microbial genomes from the northwest Pacific Ocean metagenome

**Fig. 1:**
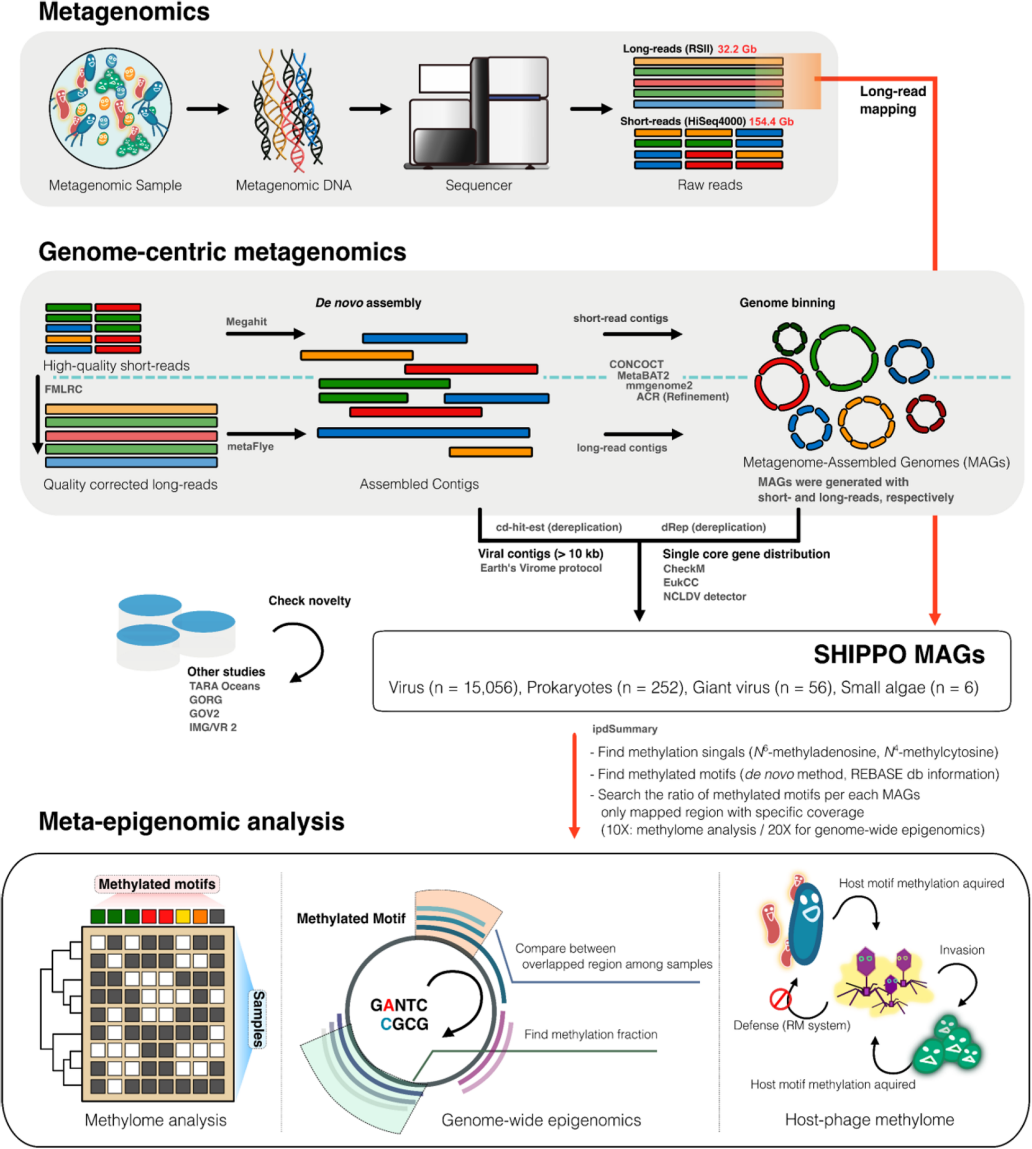
Meta-epigenome analysis scheme of ocean surface samples. A schematic overview of meta-epigenomics. Meta-epigenomics using genome-centric metagenomics from the binning approach of short- and long-read assemblies, followed by identifying the epigenetic signals of genomes from long-read mapping

**Fig. 2:**
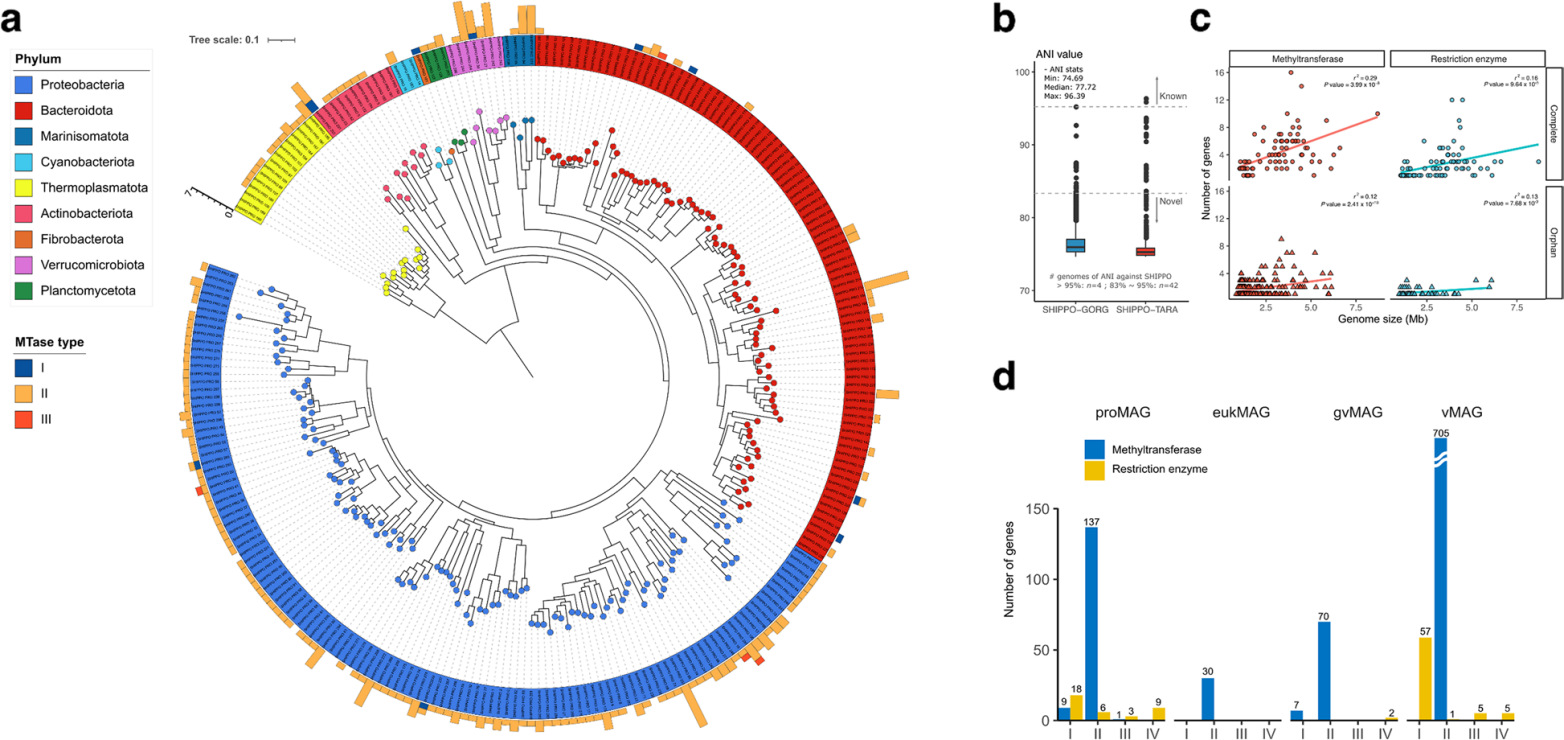
Phylogenetic tree of MAGs obtained from SHIPPO. **a** A phylogenetic tree of prokaryotic SHIPPO MAGs using core genes from Phylophlan2. A total of 252 MAGs were obtained; each bar outside the tree represents the number of methyltransferase (MTase) genes present in each MAG. **b** The distribution of restriction enzyme and MTase types from SHIPPO MAGs across kingdoms. **c** Prokaryotic SHIPPO MAGs were compared against genomes from *Tara* Oceans (TARA) and Global Ocean Reference Genomes Tropics (GORG-Tropics) datasets using FastANI. **d** The number of genes associated with the restriction-modification (RM) system is plotted against the genome size for each ocean microbiome MAG (SHIPPO MAG, TARA, and GORG-Tropics). Points are shaped depending on the type of the complete and orphan RM system. MAG: metagenome-assembled genome; SHIPPO: Shipborne Pole-to-Pole Observations

In the 2015 Shipborne Pole-to-Pole Observations (SHIPPO) project of the Korea Polar Research Institute, we conducted shotgun metagenomic sequencing using ocean surface samples from 10 stations (referred to as St2–St11) by traveling about 4000 km from the Pacific Northwest to the Bering Sea during July 22–29, 2015 (Supplementary Figure S1, Supplementary Table S1). To capture free-living organisms, we extracted genomic DNA after 0.22–1.6-μm size filtering, followed by metagenomic sequencing using short- and long-read sequencers. Extensive computational analysis was performed on all samples to reconstruct the genomes across the kingdom using a combination of individual, co-, and hybrid assembly, binning, and refinement methods (Fig. 1). This strategy allowed the recovery of a total of 15,056 viral, 252 prokaryotic, 56 giant viral, and 6 eukaryotic metagenome-assembled genomes (MAGs, specifically referred to here as SHIPPO vMAGs, proMAGs, gvMAGs, and eukMAGs, respectively; Supplementary Data 1 and 2). These proMAGs mainly consist of the bacterial phyla of Proteobacteria (*n* = 120), Bacteroidota (*n* = 88), Actinobacteriota (*n* = 11), and the archaeal phyla of Thermoplasmatota (*n* = 15) (Fig. 2a).

Short- and long-read assembly and binning strategy substantially improved the fraction of mapped metagenomic reads. Overall, the average mappability of all samples was 38.03% (std. 2.88) (Supplementary Figure S2). Most of the reads mapped to vMAGs and proMAGs, with relatively smaller representation of gvMAGs and eukMAGs. We compared our proMAGs with the *Tara* [10] and Global Ocean Reference Genomes Tropics (GORG-Tropics) [11] datasets to evaluate the novelty of our recovered genomes (Fig. 2b). Although these proMAGs and single-cell amplified genomes datasets came from a global-scale study [10, 11], only three proMAGs overlapped at the species level (≥ 95% ANI) with our proMAGs. Furthermore, 95% of the proMAGs obtained here could not be classified at the species level, even though the Genome Taxonomy Database (GTDB) [12] includes genomes of uncultured organisms derived from shotgun metagenomics and single-cell genomics. Thus, despite previous extensive ocean metagenomic binning efforts such as those undertaken on data from mega-surveys like the *Tara* Ocean Expedition [10] and the Global Ocean Survey [11], the northwest Pacific Ocean datasets from this study provide substantial novel genomic information on ocean microbiomes.

### DNA MTases in marine microorganisms

To characterize the role of MTases in ocean microbial communities, we first identified the type of MTases and their cognate REases distribution from the genome catalog established here and derived from previous ocean microbiome surveys (GORG, TARA, and SHIPPO). Of the total 5713 medium-quality proMAGs, we found 67.18% (3838) and 19.45% (1111) of proMAGs encoded one or more MTases and REases, respectively (Supplementary Data 3). Among the four MTase types—I, II, III, and IV—type II MTase was found most frequently (94.71%) in proMAGs with MTases, followed by type I (14.02%) and III (2.16%). Of all the proMAGs, only 14.77% had a complete RM system; most consisted of type I and III MTases: 76.39% and 74.70% of type I and III MTases constituted a complete RM system, respectively, whereas only 3.19% of type II MTases constituted an RM system. By contrast, most MTases (86.09%) belonged to orphan MTases, thus lacking counterpart REases, and consisted of type II MTases. To compare the genome size of ocean prokaryotes and the number of genes related to RM systems, 424 near-complete proMAGs were used. Although the number of MTase and REase correlated with genome size, the genome of proMAG with one or more RM system was significantly larger than that of proMAG with orphan MTase (Wilcoxon’s rank-sum *P* value < 2.2 × 10^−16^). In addition, in the case of proMAG with RM system, the correlation between MTase and genome size (*r*^2^ 0.29) was higher than for REase (*r*^2^ 0.16) (Fig. 2c). Overall, in the ocean microbial community, most prokaryotic RM systems suggest being composed of essential cellular mechanisms rather than the defense system. By contrast, type I and III MTases and their cognate REases typically serve as a defense mechanism through the RM system and thus are harbored in genomes of relatively large size.

Beyond MTases detected in proMAGs, a total of 959 MTases were found in the SHIPPO MAGs catalog, including all (*n* = 6) of the eukMAGs, 62.5% (*n* = 35) of the gvMAGs, 36.90% (*n* = 93) of the proMAGs, and 4.28% (*n* = 645) of the vMAGs (Fig. 2d and Supplementary Data 3). Consistent with the abovementioned results, type II MTases were the most frequently detected for all domains (Fig. 2d). All but two MTases were solitary or orphan MTases that have no counterpart REases. Eukaryotes and giant viruses had an average of 5.0 and 2.2 MTases per genome, whereas fewer MTases were found in the prokaryotic and viral genomes (1.58 and 1.09 per genome, respectively).

### DNA methylome of SHIPPO MAGs

**Fig. 3:**
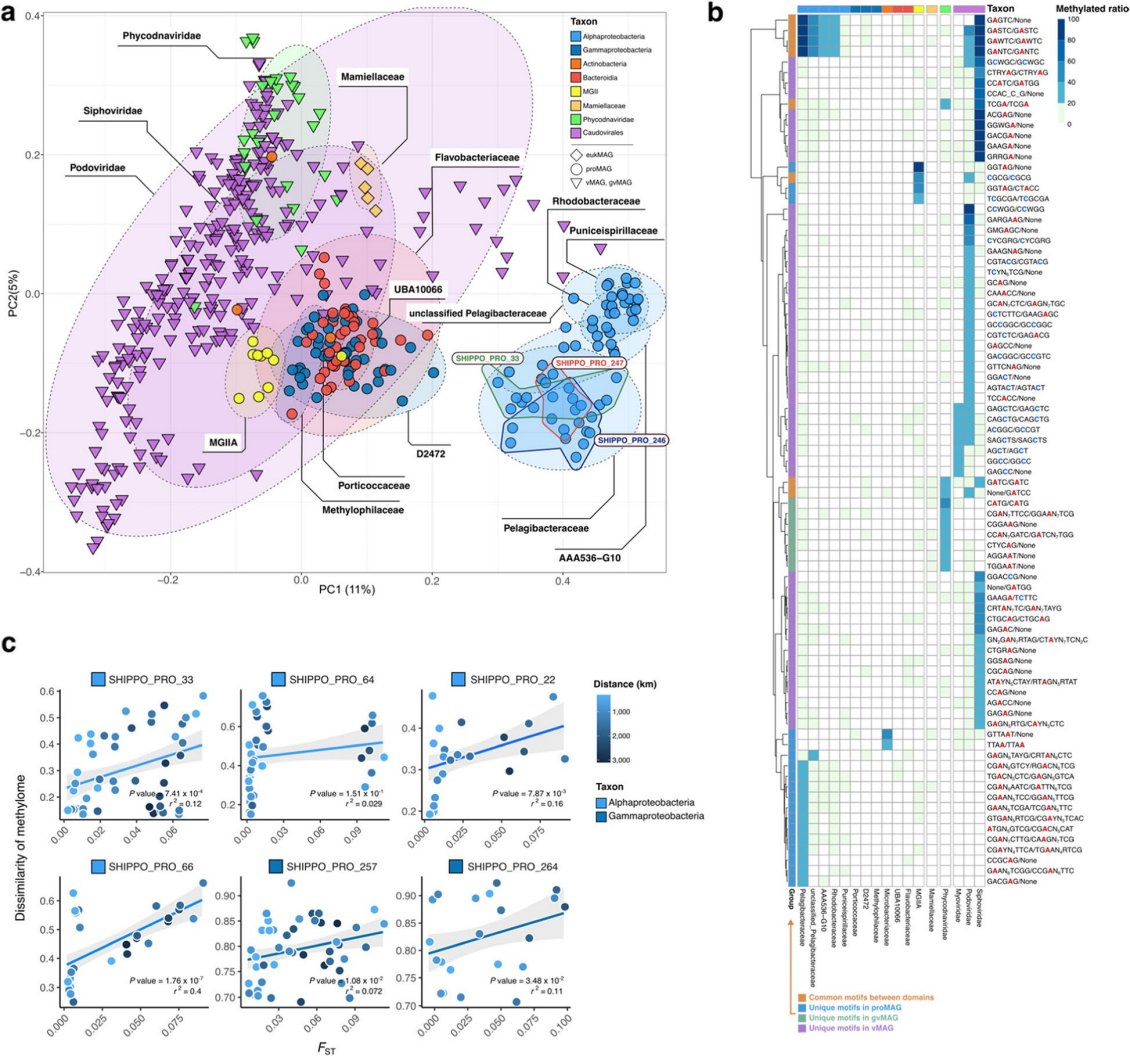
Meta-epigenomic profile of MAGs across all sampling stations. **a**, Principal coordinate analysis (PCoA) clustering by the 5-mer methylation features of Shipborne Pole-to-Pole Observations (SHIPPO) MAGs based on Kulczynski dissimilarity. Each point represents each species-level MAG in each sample. The black-dashed circles represent family-level clusters of MAGs across samples. The colored-solid lines represent species-level clusters belonging to *Pelagibacteraceae* across samples. **b** The maximum methylation ratios of motifs are represented in each MAG at the family level; the highest methylation value among all sample sites is colorized. **c** Population differentiation versus methylome across sampling stations. For the most prevalent Shipborne Pole-to-Pole Observations (SHIPPO) MAGs, scatterplots show the relationship of 5-mer methylome dissimilarity based on Bray–Curtis and population differentiation by sampling distance. MAG: metagenome-assembled genome (eukaryotic: eukMAG, prokaryotic: proMAG, viral MAG: vMAG, giant viral MAG: gvMAG); *F*_ST_: fixation index

We next studied the DNA methylation patterns of the ocean microbiome and compared DNA methylation profiles of each SHIPPO MAG across samples. We first performed a principal coordinate analysis (PCoA) based on the Kulczynski dissimilarity of 5-mer DNA methylation profiles for individual MAG-sample pairs (requiring 10× coverage with 20% genome breadth for proMAGs and gvMAGs, 10% for eukMAGs, and 60% for vMAGs; Supplementary Data 4). DNA methylation profiles were grouped clearly by domain, i.e., separating eukaryotes, prokaryotes, and virus MAGs (Fig. 3a). In particular, Alphaproteobacteria harbored distinct methylation profiles compared to all other microbial organisms, and Alphaproteobacteria proMAGs were partitioned from each other down to the family level. However, the methylation profile of 5-mers could not be distinguished at the species level, as in the example of the *Pelagibacteraceae* cluster, which consisted of SHIPP_PRO_33, SHIPP_PRO_245, and SHIPP_PRO_247. Furthermore, proMAGs belonging to Flavobacteriia, Actinobacteria, Gammaproteobacteria, and Bacteroidia were also difficult to distinguish by their methylation profile.

To identify the exact DNA methylated motif, the methylated motif information was collected via the Restriction Enzyme database (REBASE) [13]. Although additional motifs were discovered from the *de novo* motif finding algorithm MultiMotifMaker [14] across SHIPPO MAGs, most motifs were discovered in previous studies (GATC, GANTC, CGCG, VATB, underlining indicates methylation position) [15, 16]. A total of 1357 motifs were searched along the mapped region of each SHIPPO MAG. When < 20% of each motif was methylated in each genome, it was considered noise and excluded from the candidate methylation motifs. Ninety-five candidate methylated motifs were detected, of which 17 and 76 represented m^4^C and m^6^A modifications, respectively. The other two were non-palindromic motifs (CGTCTC/GAGACG, GAAGA/TCTTC) with both m^4^C and m^6^A methylation. Among the methylated motifs, 13 motifs were shared across domains. GANTC was found most frequently in several families belonging to Alphaproteobacteria and in *Caudovirales* (Fig. 3b). CGCG and GGTAG were detected in both archaeal proMAGs affiliated to MGIIA and *Caudovirales* vMAGs (Fig. 3b). The GATC motif was found in gvMAGs (*Phycodnaviridae*), *Caudovirales*, and unclassified vMAGs (Fig. 3b). In addition, there were methylated motifs unique to vMAGs, such as CCWGG and GGCC, and unique motifs were found in each family, such as TTAA (*Microbacteriaceae*), and TCGCGA (MGIIA) (Fig. 3b). The high diversity of methylated motifs in vMAG suggests the existence of many unknown MTases encoded on prokaryotic and/or viral genomes, and is consistent with a role of viral genome methylation in virus-host arms race.

To match the methylated motifs with MTases of each SHIPPO MAG, candidate MTases were searched against REBASE [13] reference MTases with recognition sequence information. Only the recognition sequences of 12 MTases were known, and except for the recognition sequences of one viral MTase, it was confirmed that the methylated motif sequences were identical to the recognition sequence information (Supplementary Table S2). For example, in the seven Alphaproteobacteria proMAGs, we could identify methylation signals from several motifs, including GANTC, GASTC, GAGTC, and CGANNNNNNAATC, but these were represented by GATNC, for which the congruent recognition sequences of the best similarity reference MTase could be found (Supplementary Table S2). There were four methylation motifs in an archaeal proMAG (SHIPPO_PRO_101), and after deduplication, two different motifs (CGCG and GGTAG) were represented (Supplementary Table S2). One congruent MTase could be found (CGCG) among these two methylation motifs, but no MTase matched the other methylation motif (GGTAG). Although the GGTAG motif was novel in that it is previously unreported, this result limited the confirmation of the archaeal novel methylation system, likely due to the fact that this MAGs does not represent a complete genome. Furthermore, except for 12 of the 1124 MTases, it was either difficult to identify the methylation profile due to the long-read sequencing depth or the lack of MTase recognition sites in the previous database made it difficult to compare between MTases and motif sequences.

### DNA methylation patterns within-population genetic diversity

Several studies of bacterial DNA methylome suggested that different bacterial strains have different methylome patterns, even within species [8, 17, 18]. These changes can be caused by the presence of MTases or by phase-variable MTases that respond to changes in the environment [19]. However, microbial DNA methylation changes in complex environments have not yet been measured directly; therefore, we analyzed intra-species DNA methylation variation at the sampling station. The fixation index (*F*_ST_) was used to compare the similarity in the population differentiation between samples. proMAGs with low base-pair coverage were excluded because the *F*_ST_ had to be calculated by the allele frequencies within the species. *F*_ST_ was calculated for dominant proMAGs using a mapping region that overlaps at least 40% breadth with 10× depth in all samples with short reads. The dissimilarity of methylomes was calculated by the methylation frequency of 5-mer nucleotides in a genome. Therefore, to compare the DNA methylation changes for different sampling stations, only six species-level proMAGs were fulfilled by the sequencing coverage of long- and short reads. In five of the six proMAGs, DNA methylome differences and population differentiation across samples correlated significantly, regardless of the distance between sampling stations (Pearson correlation, *P* value < 0.05; Fig. 3c). A significant correlation was also found in Gammaproteobacteria proMAGs that showed no specific methylated motif (weak methylated motifs ratio < 10%). These results may indicate that when multiple strains with different methylation profiles of motifs are present in the environment, they affect the methylation pattern at the species level.

### Genome-wide DNA methylome of Pelagibacter in environmental samples

Next, we analyzed the methylation pattern of dominant proMAGs at the single-nucleotide level to investigate in detail the environmental-dependent changes in methylation. Identifying the single-nucleotide-level DNA methylation from a genome-wide perspective required a relatively deeper and wider read coverage. Four proMAGs (SHIPPO_PRO_33, SHIPPO_PRO_246, SHIPPO_PRO_64, and SHIPPO_PRO_101) were covered over > 40% of the breadth of the genome at 20× depth per strand. Of these, only a SHIPPO_PRO_33 proMAG affiliated with *Pelagibacter* overlapped at 65.61% of the genome breadth in all 10 samples. The average breadth of the genome coverage with 20× per strand of SHIPPO_PRO_33 was 90.93% (std. 7.40). The in-depth overlapped coverage of long-reads enabled a comparison of methylation patterns between samples for this specific *Pelagibacter* proMAG.

To compensate for the lack of sequencing depth in the genome between samples, we focused on the genome-wide epigenetic analysis of the *Pelagibacter* proMAG (SHIPPO_PRO_33; 12 contigs; 87.30 completeness, 3.79 contamination). Only the nucleotide positions of the overlapped regions that could measure methylation in all samples were compared. A total of 2,494 GANTC motifs were detected on both strands of the SHIPPO_PRO_33 genome, and only 1719 GANTC (68.93%) were included in the overlapped region (the average depth of reads was 112.85×, std. 60.56) for measuring the methylation at 10 sampling stations. The methylation rate of GANTC varied in the range 71.31–94.77% depending on the sample, of which 2.44% (42 sites) remain unmethylated in all samples. In most cases, unmethylated sites were frequently observed in intergenic regions (66.67%), including regulatory regions (16.70%) (Supplementary Table S3). The GANTC position of one of these seven unmethylated positions of the regulatory region contains the *sufE* (K04488) regulatory region of the Suf (sulfur-forming) system. In addition, it was found to be related to genes essential for the cellular mechanism of *Pelagibacter*, particularly heat shock protein (*groEL*) and large subunit ribosomal protein.

**Fig. 4:**
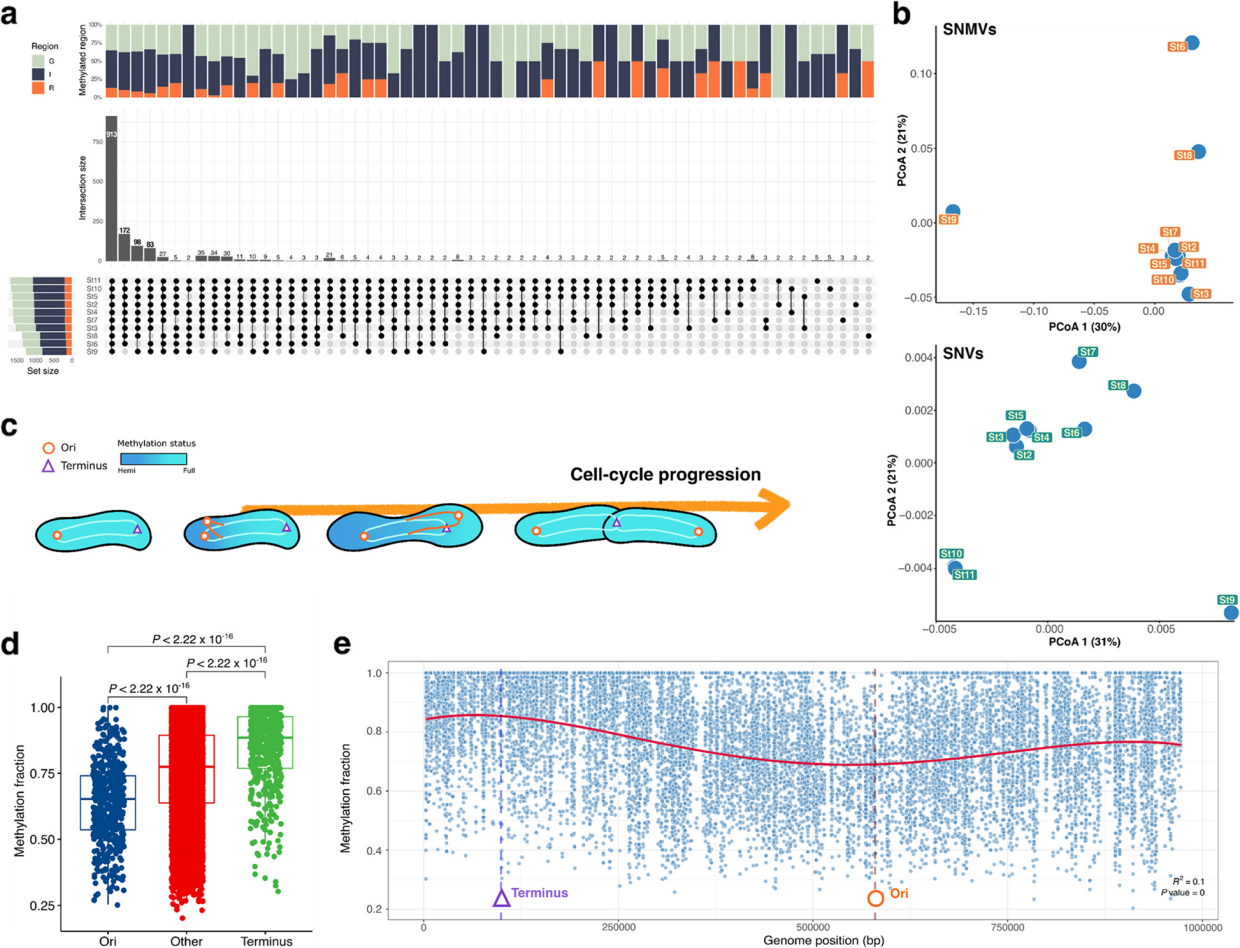
Genome-wide epigenetic analysis of *Pelagibacter* MAG across samples. **a** The UpSet plot compares GANTC methylation at each genome position on a *Pelagibacter* MAG (SHIPPO_PRO_33) across samples. More than 0.5 of the methylation fractions were considered methylated at each genome position; the color of each bar depends on the genic (G: green), intergenic (I: navy), and regulatory region (R: orange). The column bar indicates the intersection of the number of methylation positions on the MAG across samples. The left bar represents the total number of methylated positions on the MAG for each sample. **b** Principal coordinate analysis (PCoA) clustering by SNMV and SNV based on the Bray–Curtis distance on overlapped regions for all samples. **c** A model of the methylation pattern according to the cell-cycle progression in Alphaproteobacteria. **d** The methylation fraction comparisons of the GANTC motif between genomic regions of *ori* (replication origin), *ter* (replication terminus), and other regions. **e** The genome-wide distribution of methylated fractions for the GANTC motif indicates the trend of cell-cycle progress throughout the genome. MAG: metagenome-assembled genome; SHIPPO: Shipborne Pole-to-Pole Observations; SNMV: single nucleotide methylation variation; SNV: single nucleotide variant

The DNA methylation signal at the nucleotide resolution is calculated from the pooled interpulse duration (IPD) ratio in separate molecules for each genomic locus. Due to the often-found epigenetic heterogeneity [20, 21], these aggregated methylation signals indicate the methylation of cell fractions at the nucleotide level (hereafter referred to as methylation fraction). The methylation fraction of GANTCs was observed in heterogeneity across samples, particularly in St6, St8, and St9. The differences in the methylation fractions on nucleotide sites were referred to as single nucleotide methylation variation (SNMV). Compared to other samples, the number of unmethylated motifs was higher in St6, St8, and St9 and showed different SNMV patterns at each nucleotide position (Fig. 4a). By contrast, all samples, except for St6, St8, and St9, had similar SNMVs to each other and were more closely clustered, regardless of latitude (Fig. 4b). For example, pairs St2 and St3, and St10 and St11, distanced geographically by about 20° latitude and 3000 km distance, were grouped closely through PCoA (Fig. 4b). In particular, differences in strain-level composition (single nucleotide variants; SNVs) were found between these two groups, but in SNMVs, these groups were clustered together (Fig. 4b). These results indicated that DNA methylation at the single nucleotide level differed under environmental conditions regardless of strain composition, which suggests that dynamic cellular events occur among various *Pelagibacter* in northwest Pacific Ocean surface waters.

### Active replication of Pelagibacter refelected by MTase activity

Several MTases, such as CcrM, which methylates the GANTC motif, are involved in the cell-cycle regulation in Alphaproteobacteria [22]. When re-analyzing previously published genomic data capturing methylation status of Cand. P. Giovannoni NP1 chromosome during exponential growth [23], we noticed a gradual decrease of the genome-wide methylation fraction of GANTC from the replication origin (*ori*) to replication terminus (*ter*) (Supplementary Figure S3). The proposed underlying mechanism [24] is thought to be linked to delayed methylation of the daughter strand: as chromosomal replication proceeds, the methylation status of the parental strand is maintained, whereas GANTC remains unmethylated when a new daughter strand is generated through a replication fork (hemimethylated) (Fig. 4c). As methylases, such as *ccrM* [22]*,* are typically only expressed at the end of chromosome replication, the chromosome remains hemimethylated until the end of replication. When DNA methylation signals are pooled from separate molecules for each genomic locus, the methylation fraction may result in relatively lower values near the *ori* due to the ratio of hemimethylation to full methylation following different DNA replication instances for different cells in the exponential phase (Supplementary Figue 3). The same pattern of a lower methylation fraction in the *ori* region of the chromosome was observed with the *Pelagibacter* proMAG (SHIPPO_PRO_33) across several samples under natural environmental conditions (Fig. 4d, e and Supplementary Figure S4). This suggests that some *Pelagibacter* strains are also in an exponential growth phase in natural marine environments, and that GANTC methylation may also be involved in cell cycle regulation for marine *Pelagibacter* strains.

### DNA methylation of viral genomes

**Fig. 5:**
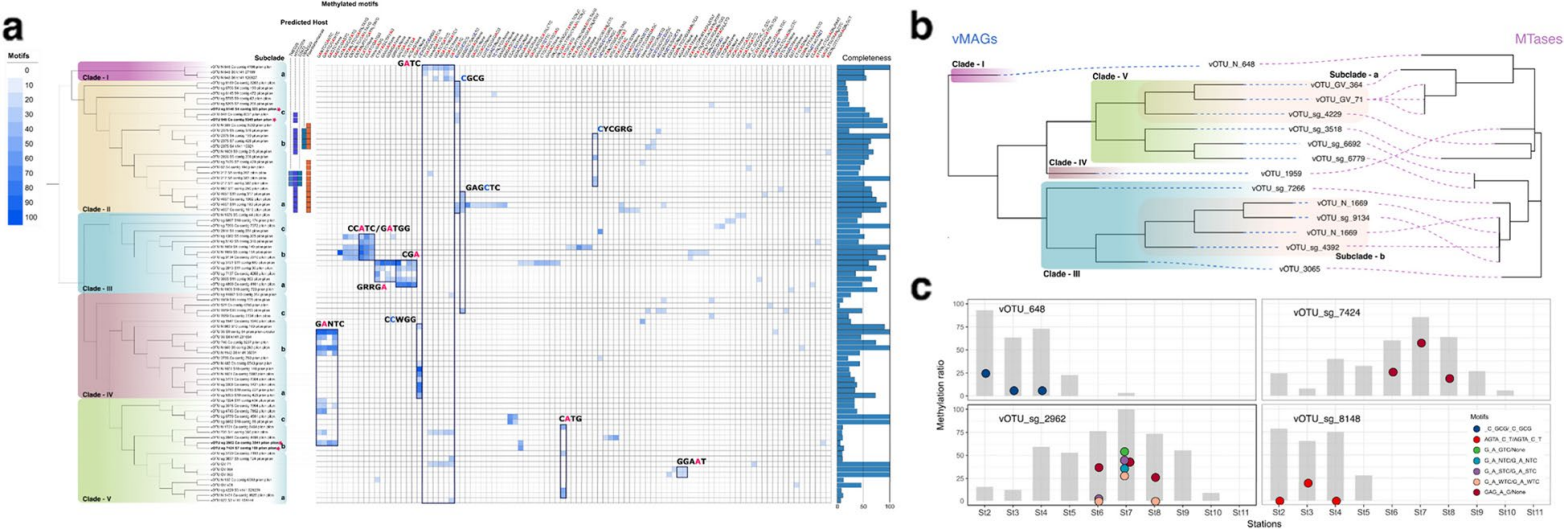
DNA methylation of the viral genome. **a** The methylome of 83 prevalent viral Shipborne Pole-to-Pole Observations (SHIPPO) of metagenome-assembled genomes (vMAGs) is indicated by the heatmap with their phylogeny from genome similarities. The star represents vMAGs in **c**. **b** Phylogenetic comparison of the 14 vMAGs harboring MTase and its MTase genes. **c** Changes in vMAG methylation profiles were measured at three sampling stations. Circles represent the methylation ratio of each motif, and bars represent the read mapping breadth of viral genomes with 10× depth

We only observed methylation patterns in 83 vMAGs associated with 67 vOTUs (viral operational taxonomic units) in a total of 15,056 putative viral genomes. The 83 vMAGs grouped into five major lineages based on pairwise genome similarity (Fig. 5a). Most of the clades were composed of vMAGs belonging to the *Caudovirales* order, except for clade V which showed genomic similarity to high-quality gvMAGs, and is likely composed of genome fragments related to *Phycodnaviridae*. Within the individual clades, there were typically two or three distinct subclades with outgroups, and some methylation patterns were shared between subclades. For instance in clade-III, specific adenine methylation motifs were found within subclades, which were not shared with other clades. Although these subgroups are phylogenetically related, the two different methylated adenine motifs (GRRGA/None and CCATC/GATGG) did not overlap and represent entirely different motif patterns. Cytosine methylation was dominant throughout clade-II, and methylation was observed in CGCG, GAGCTC, and CYCGRG motifs according to subgroups. On the other hand, both adenine and cytosine methylation were found in clade-IV, and specific methylation was indicated in the GANTC and CCWGG motifs, respectively, depending on the clade. In addition, the methylation in the GATC motif was consistent in clade-I and, together with the motifs found in clades-IV, suggests that it may be associated with bacterial methylation system as a methylation motif frequently found in Proteobacteria. For clade-V, adenine methylation was detected, as previously reported for *Phycodnaviridae* [25]; in our study, these were found on GATC and CATG motifs. These two methylated motifs were observed spanning genomes belonging to *Phycodnaviridae*, and importantly the same motif has been previously reported in some of their green algae hosts [15].

To investigate how viral methylation patterns are associated with MTase genes, we next evaluated the distribution of MTases according to the phylogenetic distribution of vMAG clades. Of 83 vMAGs, we found only 14 vMAGs encoded MTase genes, all of which belonged to the Type II MTase. Most MTases showed phylogenetic consistency with the vMAGs (Fig. 5b), along with consistencies in the methylated motifs. For instance, four vMAGs in the clade-IIIb, all methylated to the CCATC/GATGG motif were found to encode closely related MTases, yet these MTases had less than 50% similarity to compare within the REBASE. This lack of similarity to characterized MTAses was a common observation for vMAG-encoded MTAses, and there was thus only one instance of methylated motifs consistent with the predicted recognition sites based on the closest existing Mtases, for vOTU_N_648 in Clade-I. This suggests that most viral MTase systems are still poorly characterized, and we expect that methylation motifs for these novel MTases could be predicted based on the methylation patterns and phylogenetic distributions of their corresponding vMAGs.

### Possible implication of methylation in SAR11 phage-host interactions

**Fig. 6:**
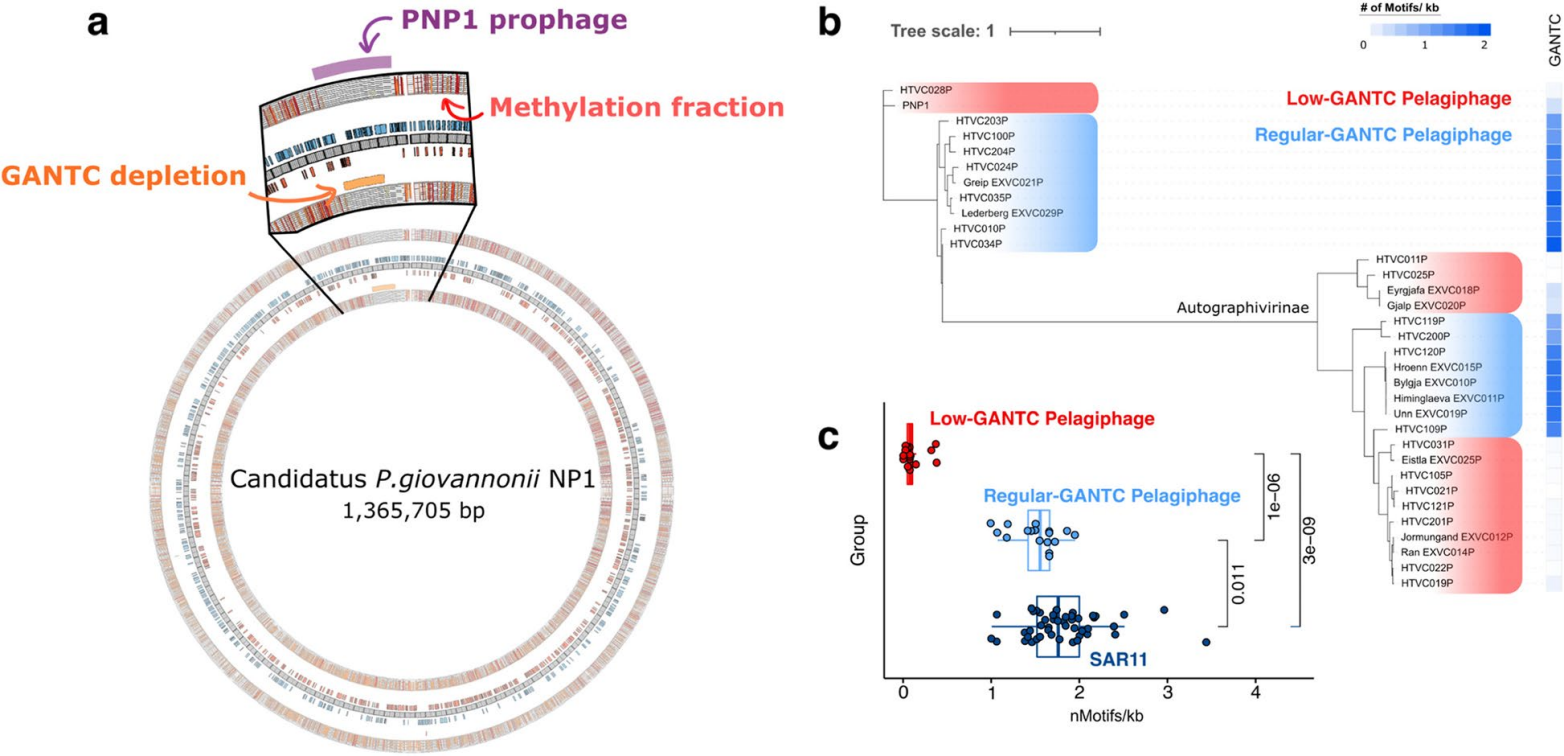
Genomic characterization between *Pelagibacter* and pelagiphage according to the GANTC motif. **a** Distribution of GANTC motif methylation in both strands of *Cand.* Pelagibacter Giovannoni NP1 genome. The inner blue and red bars indicate the coding sequence region of the genome. The methylation fraction bar for each strand is red for values ≥ 0.9, orange for ≥ 0.6, and bright yellow for ≥ 0.3. The prophage region of the genome is highlighted by a purple bar from Pleška et al. [26]. The GANTC motif depletion region is marked through a multiscale signal representation (MSR) analysis. **b**, Phylogenetic tree of pelagiphages of the head–tail connector protein. Red boxes represent the GANTC depletion subgroups of pelagiphage. **c** The GANTC motif density comparisons between genomes of *Pelagibacter* and pelagiphage

Most GANTC positions were methylated throughout the *Cand.* P. Giovannoni NP1 genome (Supplementary Figure S3), and this methylation mark is thought to be related to cell cycle regulation (see above). However, uneven methylation patterns were observed in specific regions of the genome due to the lack of GANTC motifs in the proviral region (Fig. 6a). This raised questions about the potential use of the same methylation mark as a defense system, and the evasion of this defense system by pelagiphages (phages infecting SAR11 bacteria) at point in the evolutionary history of the phage-host pairs.

We next reconstructed the phylogeny of 33 publicly available pelagiphage genomes based on a head–tail connector protein and compiled the frequency of GANTC motifs in the same genomes (Fig. 6b). Surprisingly, about half of the pelagiphage genomes showed a clear depletion in GANTC motif, and the calculated phylogenetic trees clearly showed that the phages clustered according to the density of GANTC motifs in their genome (Fig. 6b, c). This difference was particularly observed in the subfamily *Autographivirinae* of the family *Podoviridae*.

## Discussion

Several studies have investigated DNA methylation patterns of microorganisms to understand the epigenetic process regulating functional repertories, virulence, and cell cycle through laboratory cultures. Due to advances in sequencing technology, the epigenome of individual prokaryotes has been studied extensively in recent years, but the overall patterns of direct DNA methylation in the environment has rarely been explored. There are three main reasons why epigenetic studies have not been conducted frequently through metagenomic studies. First, until recently, it was difficult to obtain representative genome from environmental samples due especially to the high diversity of environmental microorganisms. Second, it is still challenging to measure DNA methylation in-depth as it requires relatively expensive long-read sequencing. And finally, there is a lack of pipelines and guidelines for community-wide methylome analysis. To address these limitations, we present here a short-read and long-read hybrid analysis approach yielding high-quality de novo genomes and genomic methylation information at the community scale. Importantly, beyond unique methylation information, the quality-corrected long reads also allowed us to obtain genomes that are difficult to reconstruct otherwise due to high strain diversity, including a novel *Pelagibacter* genome. This analysis framework enables us to describe the distribution and potential biological impacts of DNA methylation in marine microorganisms, that have not been previously interpreted from an environmental perspective.

RM systems are ubiquitous (i.e., they are found in about 90% of the bacterial genomes [27]) and typically act as a defense mechanism to distinguish between self and non-self DNA. However, upon investigating RM system diversity within the ocean microbial communities from the large-scale study of *Tara* Ocean and GORG to our research, complete RM systems were mainly found in RM I and III. In contrast, the type II RM system was not, as most were formed by orphan MTases without cognate REases. Unlike the other types, the type II RM system is known as a key strategic defense system of prokaryotes against diverse invasive foreign DNA (e.g., plasmids or phages) because the recognition sites of MTase and REase are specifically similar [28]. Particularly, type II RM systems have constant opportunities for genetic transfer through horizontal gene transfer and homologous recombination [29], and also exhibit subsequent addictive characteristics that can be selfishly transmitted in populations [30]. Among them, only MTase, not REase, typically remained an orphan, which may be explained by the degradation or inactivation of congruent REase after the acquisition of the RM system by horizontal acquisition [31, 32]. In particular, the loss of one component from a horizontally-acquired type II RM system may be driven by the selective pressure of genomic streamlining in the pelagic environment. Alternatively, as part of a horizontal gene transfer process of the RM system, MTase may be transferred before REase to protect host DNA from cleavage [33]. Regardless of the underlying mechanisms, it appears that type II MTases are much more frequently detected as orphan MTases in the SHIPPO dataset. In these cases of orphan MTase, DNA methylation is likely to be used to as regulator of cellular mechanisms rather than as defense mechanisms of exogenous invasive DNA, because this latter role would require the REase component.

After investigating the potential roles of methylation globally, we next studied the methylation patterns for the most dominant organism, *Pelagibacter*, in more detail. As mentioned above, the MTases of most marine microorganisms were found without counterpart REases, and conserved MTases were also observed without counterpart REases in the four *Pelagibacter* proMAGs. In particular, the *Pelagibacter* proMAGs showed that the methylated motif (GANTC) and the recognition motif of their MTase were consistent. The GANTC motif in the *Pelagibacter* proMAGs was globally uniformly distributed throughout the genome (Shapiro–Wilk test: genic > 0.05, intergenic > 0.05, and regulatory > 0.05). For bacterial MTases involved in gene regulation, methylated motifs are frequently located upstream of regulated genes [28]. In this study, we also found that GANTC was enriched in the intergenic region of *Pelagibacter*, indicating that GANTC favors an epigenetic role (*P* value = 0.05, Supplementary Figure S5).

Although most motifs are known to be methylated throughout prokaryotic genomes [16], some motif sites have been found that remain unmethylated [28, 34, 35]. These unmethylated sites may be due to competitive binding between MTases and regulatory proteins due to epigenetic regulation [36, 37]. Therefore, as an epigenetic role was expected from the GANTC distribution within *Pelagibacter* proMAG (SHIPPO_PRO_33), the methylation status of different GANTC loci in this genome was explored to identify potential epigenetic unmasking [17, 28]. Overall, unmethylated GANTC motifs were observed across multiple stations and loci (Fig. 4a, b). Particularly, samples from St6, 8, and 9 showed a higher number of unmethylated sites, which lacked a clear explanation when considering the geographic, temperature, and salinity gradient in the dataset. Understanding the epigenetic regulation of all GANTC loci in a genome-wide manner remains an important challenge. However, combining metatranscriptomics and metaproteomics with environmental metadata in a larger dataset may help to further elucidate these mechanisms. Across the SHIPPO dataset, the only GANTC positions that were broadly unmethylated were observed in intergenic and likely regulatory regions, including regions upstream of the *sufE* (K04488) gene from the Suf (sulfur-forming) system. This gene is part of the SufSE complex with *sufS* and is involved in the transport of sulfur to the sulfur mobilization protein. Along with *sufS*, this gene was also significantly more highly expressed in the absence of dimethylsulfoniopropionate (DMSP) in sulfur-limited conditions in the laboratory [38]. We also observed unmethylated GANTC in the regulatory region of *groEL* (chaperone gene, K04077), which is also more highly expressed in sulfur-restricted conditions [38]. Furthermore, because proteins are constantly being degraded due to exposure to environmental stresses, metaproteomics revealed that *groEL* of SAR11 was among the most abundant proteins on the ocean surface, and *groEL* genes are thus likely to be highly expressed [39]. Moreover, based on previously published data, we found that most GANTC motifs (99.86%, 3477/3482) were fully methylated (both strands were methylated) under DMSP-rich laboratory culture conditions of *Candidatus* Pelagibacter Giovannoni NP1 [23], which is most closely related to SHIPPO_PRO_33 (ANI ≥ 92.36). *Candidatus* Pelagibacter ubique, which harbors a streamlined genome, typically requires a source of exogenous reduced sulfur compounds such as DMSP for growth [26]. Natural DMSP levels in the surface seawater range from 1 to 100 nM [40–42], which constitutes a significantly oligotrophic environment relative to DMSP-rich laboratory conditions (10 μM) for culturing *Pelagibacter*. Therefore, the differences in unmethylated GANTCs between environmental and laboratory settings may reflect the methylation-based epigenetic regulation of nutrient acquisition.

Viruses primarily use DNA methylation to counter host defense systems, such as the RM system, although some also exploit methylation to signal a transition in their status from a latent to a lytic state [43]. The methylation of viral DNA is also inherently transient as it is either derived from viral-encoded MTase or the host's MTase and can be demethylated via passage through an MTase-free host. Viruses most often use their own MTases as a counter-defense and as a signal to initiatelysis state and DNA packaging [44, 45]; however, incongruences were observed in our study between the methylome of the viral genome and the recognition motif of the predicted MTase. Therefore, DNA methylation of the viral genome in the marine environment is most likely a marker left behind from a previous host, rather than driven by virus-encoded MTases. To support the hypothesis, we further analyzed the DNA methylomes of the viral genomes detected at multiple sampling stations, and confirmed that methylation patterns and loci differ between geographic locations for individual viruses (Fig. 5c). Although further studies are needed to understand the biological and ecological significance of DNA methylation in viruses in the real world, an explanation for our results is that a substantial amount of methylation in virus genomes derive from their host machinery, and different hosts altered the virus methylome with MTases that recognize different motifs.

Finally, we noticed that in the eight proMAGs of the SHIPPO catalog spanning the order Pelagibacterales, their genomes lacked all genes associated with typical phage defense mechanisms including CRISPR and RM systems. Consistent with our results, there have not been previously reported RM systems in the SAR11 genome, except for a few common defense systems on some SAR11 genomes [46]. Despite the absence of identifiable RM system in our SHIPPO *Pelagibacterales* proMAGs, we did observe a clear GANTC density bias in the genome of some known pelagiphage clades. This raises the possibility that methylation marks may have been used as anti-phage defense in the past, or that some strains within or related to the SAR11 clade do use RM systems as phage defense, in which case the difference in GANTC frequency would be related to differences in host range between phage clades.

## Conclusion

Genome-centric metagenomics leverages reconstructed genomes spanning multiple kingdoms -including viruses, prokaryotes, and eukaryotes—to investigate the biological processes occurring in natural microbial communities. Here, we analyzed community-wide and single-nucleotide level epigenetics in the oceanic microbial communities, and the approach proposed herein provides a roadmap for how to analyze DNA methylome changes across different environments. Additionally, successful genome reconstruction using long- and short-reads allowed for the inference of phage-host-related methylation and co-evolutionary histories. Overall, our findings illustrate how genome-wide epigenetic analysis and phage-host-related methylation, along with the establishment of global metagenome-methylation databases, can help characterize microbiomes.

## Methods

### Sample collection and shotgun sequencing

Surface seawater samples (7 m depth) were collected at 10 stations (referred to here as St2–St11) along a transect of 44–63°N latitude (approximately 4000 km) in the northwest Pacific Ocean during the R/V *ARAON* cruise of the Korea Polar Research Institute (KOPRI) for a Shipborne Pole-to-Pole Observations (SHIPPO) project in July 2015 (Supplementary Figure S1 and Supplementary Table S1). Onboard, 20 L of seawater at each station was immediately filtered through a 147-mm GF/A glass microfiber filter (Whatman, Maidstone, UK) and subsequently through a 147-mm, 0.22-μm membrane filter (Millipore, Billerica, USA) using a peristaltic pump. These filters were stored at -80°C until DNA extraction. The filters were chopped and dissolved in sucrose-Tris-EDTA buffer (STE buffer; 0.74 M sucrose, 1 M Tris pH 8.3, 0.5 M EDTA, and distilled water). Next, samples were treated with lysozyme (5 mg/mL Tris-HCl), followed by 10% SDS and proteinase K (20 mg/mL). Genomic DNA was extracted using a DNeasy Blood & Tissue Kit (QIAGEN, Hilden, Germany) following the manufacturer-provided protocol. Long-read shotgun sequencing was conducted using the Pacific Biosciences (PacBio) RSII sequencing system (Menlo Park, CA, USA) with P6-C4 chemistry at DNA Link (Seoul, Korea) according to the MagBead OneCellPerWell v1 protocol. Each DNA sample was sequenced in continuous long-reading (CLR) mode with a library size of 5 kb, and three single-molecule real-time (SMRT) cells were used per sample (a total of 32.2 Gb was generated with read average of 797.9 bp length). In addition, for short-read shotgun sequencing, libraries were constructed by TruSeq Nano and sequenced using an Illumina HiSeq 4000 system with paired-end reads (a total of 154.4 Gb was generated with read average of 151 bp length) at Macrogen (Seoul, Korea).

### Read preprocessing and contig assembly

Short-reads were preprocessed with FaQCs (v. 2.09; the default option with -*q* 20) [47], and a duplicated read removal process was performed using FastUniq [48]. Quality-filtered reads of each sample were assembled into contigs using MEGAHIT (v. 1.1.3) [49] in “meta-sensitive” mode. In addition, reads of all 10 samples were assembled into contigs through co-assembly with the same options as above.

Long-reads were preprocessed with a hybrid error correction approach using FM-index Long Read Corrector (FMLRC; v. 1.0.0) [50] with quality-filtered short-reads. After preprocessing, corrected long-reads of each sample were assembled using Flye (v. 2.4.1) [51] with “meta-Flye” and “PacBio corrected reads” options. Co-assembly was also performed on all 10 samples with the same options as above. Furthermore, assembled contigs from long-reads were polished using Pilon (v. 1.23) [52] twice.

### Genome binning of the northwest Pacific Ocean

Contig coverage was calculated using only short reads, which consisted of a uniform length (151 bp) with the Burrows-Wheeler Aligner tool (BWA-MEM v. 0.7.17) [53]. Next, contigs from individual assembly and co-assembly were binned separately using CONCOCT (v. 0.5.0) [54] and MetaBAT (v. 2.12.1) [55], respectively. Additionally, a manual binning process was performed with mmgenome2 (https://github.com/KasperSkytte/mmgenome2). Then, these bins were refined by the integrative *k*-means clustering approach (ACR: https://github.com/hoonjeseong/acr) using contig coverages.

For prokaryotes, the quality of genome bins was estimated using CheckM [56]. Only bins with better than medium quality (completeness > 50% and contamination < 10%) [8] were referred for metagenome-assembled genomes (MAGs) and used for further analysis. All MAGs were dereplicated at 99% average nucleotide identity (ANI) value using dRep [57] with the options “-comp 50-con 10” and then dRep was re-run with “-sa 0.95” (95% ANI value clustering) to screen representative species MAGs. To improve the quality of short- and long-read sequencing, a reassembly process was performed on only high-quality MAGs (HQ MAGs, completeness > 90% and contamination < 5%). Quality-filtered short- and long-reads were mapped against HQ MAGs using bowtie2 [58] and PBAlign (https://github.com/PacificBiosciences/pbalign). Then, mapped reads were retrieved using “samtools view” with -f 65, -f 129, and -F 4 options for forward, reverse, and unpaired long reads, respectively [59]. Each HQ MAG was reassembled with the hybrid assembly approach using Unicycler [60]. The obtained prokaryotic MAGs (referred to as SHIPPO PRO MAG) were designated as SHPPO_PRO_XX and classified using the Genome Taxonomy Database Toolkit (GTDB-Tk) [11]. The phylogenic tree was constructed by PhyloPhlAn2 [61] and visualized by iTOL v. 6 [62].

For eukaryotes, eukaryotic bin classification was performed for those > 5 Mb in size by EukRep [63]. Afterward, a detailed quality evaluation was performed through EukCC [64], and eukaryotic MAGs were selected according to the criteria of “Completeness-5*Contamination > 50.” In addition, the MAG with the highest score (Completeness-5*Contamination + 0.5*log(N50)) in the group within ~ 99% of the ANI value was selected as the representative eukaryotic MAG. Furthermore, species-level eukaryotic MAGs were chosen by the highest scores within ~ 95% ANI cluster. The eukaryotic MAGs (referred to as SHIPPO EUK MAG) were designated as SHPPO_EUK_XX, and their taxonomy was classified by phylogenetic topology with MAGs from Delmont et al. [65]. Phylogenetic analysis was processed with two DNA-dependent RNA polymerase (RNAP-a and RNAP-b) subunits. The concatenated protein sequences of RNAP-a and -b were aligned by MAFFT alignment (v. 7.402, FFT-NS-i algorithm with default option) [66], and gap sequences were trimmed using trimAl (v. 1.4) [67] with the parameter -gt 50. The phylogenetic trees were calculated by IQ-TREE (v. 1.6.12) [68] using the following parameters: -m LG + F + R10 -alrt 1000 -bb 1000; LG + F + R10 model, 1,000 replication of the Shimodaira-Hasegawa (SH)-like approximation likelihood ratio (aLRT) and ultrafast bootstrap approximation (UFBoot) [69].

The viral contigs were detected using VirSorter (only categories 1, 2, and 3) [70] and the Earth's Virome protocol [71] from all contigs through individual and co-assemblies. CD-HIT-EST [72] clustering was processed with 99% identity to remove duplicate viral contigs, and only > 5 kb contigs were subsequently used. Then, viral sequences were clustered as “population” with 95% ANI, which are currently defined as species-level taxonomy in viruses (viral operational taxonomic units: vOTUs)—via all-vs.-all pairwise BLASTn [73] using previous viral genome datasets (the Earth's Virome protocol [74] and the Global Ocean Viromes 2.0; GOV2.0 [75]). vOTUs were clustered with 95% identity across ≥80% aligned fraction, and at least one hit > 1 kb in length. Groups with previous study datasets were designated as vOTU_XX, groups of two or more viral contigs only in the SHIPPO set were designated vOTU_N_XX, and singletons were designated vOTU_sg_XX (referred to as SHIPPO vOTU). For giant viruses, dereplicated viral contigs were binned using MetaBAT2 and filtered using NCLDV_detector. Then, giant viral bins were further filtered with the same conditions as in Schulz *et al.* [76] through the quality assessment using the copy number of NCOVGs. These giant viral MAGs (referred to as SHIPPO GV) were designated as vOTU_GV_XX. Their ANI values were compared and then selected for representative strain- and species-level genomes with the completion and duplication ratio of NCOVGs. Next, the quality of vOTUs was assessed using CheckV [77] and divided into four groups: “complete” (0.02%, *n* = 4), “high quality” (2.15%, *n* = 415), “medium quality” (5.45%, *n* = 1052) and “low quality” (92.02%, *n* = 17,758). Taxonomic classification of vOTUs was assigned by Demovir (https://github.com/feargalr/Demovir) with the comparison of amino acid sequence homologies to a viral subset of the TrEMBL database. Phylogenetic analyses were processed based on the Dice distance using sequence similarity comparisons of each contig using all-vs.-all tBLASTx (≥30% identity, ≥30 amino acids, and an e-value ≤0.01). The Dice distance between contigs A and B (D_A,B_) was calculated as 1 − (2 × AB/AA + BB), where AB is the bit-score sum of all hits between genomes A and B. AA and BB are the bit-score sums of all hits from genomes A and B, respectively, compared to themselves. Then, we obtained a phylogenetic tree from the distance matrix via a neighbor-joining algorithm.

A total of 252 dereplicated proMAGs (99% average nucleotide identity; ANI) with ≥ 50% completeness and < 10% contamination remained (average completeness: 78.80 ± 14.09, and contamination: 2.76 ± 2.37), 105 originated from the individual binning and 147 from the co-assembly binning. Forty-seven had > 90% completeness and < 5% contamination (near-complete), of which only three were high-quality MAGs that fit the Minimum Information about a Metagenome-assembled Genome (MIMAG) criteria [78] including rRNA and tRNA.

### ANI comparison with reference datasets

To ascertain the novelty of SHIPPO MAGs, we collected two genome databases that derived ocean metagenomics (the *Tara* Oceans survey [10] and the Global Ocean Reference Genomes Tropics survey; GORG-Tropics [11]) for comparison with our data. The quality of MAGs from *Tara* Oceans and single-cell assembled genomes (SAGs) from GORG-Tropics were assessed using CheckM [56]. Genomes that were not at least medium-quality were excluded from subsequent analysis. We calculated the ANI values of SHIPPO MAGs for 957 MAGs and 4741 SAGs, respectively. Intra- and inter-species boundaries were differentiated with 95% and 83% ANI values, respectively, as previously studied.

### Sample mapping

Each short-read of a sample was subsampled at 49 million paired reads (minimum sequencing throughput among samples) and mapped to the SHIPPO MAGs using Bowtie2 (v. 2.3.4.3) [58] with “--very-sensitive” parameter in end-to-end mode. Additionally, to avoid overestimating the read alignment, we excluded reads whose alignments score was < -20 (tag AS:i). The percentage of mapped reads to SHIPPO MAGs (mappability) was calculated by dividing the aligned reads by subsamples (49 million) paired reads in each sample.

### Diversity calculation

To calculate species-level diversity, mapped reads to species-level SHIPPO MAG were further filtered with the read alignment identity (≥95%) using pysam. Macrodiversity was calculated using Shannon diversity by the skbio diversity Python module (http://scikit-bio.org/) for each sample. Before calculating species microdiversity, we observed how many positions within the genome were shared by all samples with sufficient read depth. We only used MAGs with shared mapping positions across all samples wider than 20% of the genome breadth (60% for the viral contig) with ≥10 read depth. Then, single nucleotide variants (SNVs) were called with the following command: “samtools mpileup -l [overlapped region bed file] -0u -C 50 -Af [SHIPPO fasta] [bam files] | bcftools call -vm0 -v -o [output vcf],” where “[overlapped region bed file]” is the bed format of the overlapped region from read-mapping, "[SHIPPO fasta]" is the fasta file of species-level SHIPPO MAG sequence, “[bam files]” are all bam files that result from read mapping to SHIPPO MAG sequence, and “[output vcf]” is the output vcf file of variant information. Each SNV was considered single nucleotide polymorphism (SNP) loci by classical definition as having an alternative allele supported by a frequency > 1% and at least four reads. The microdiversity (nucleotide diversity, π) of each genome was calculated using an equation from Schloissnig et al. [79]. Furthermore, the fixed index (*F*_st_)—a measure of the population differentiation between samples—was calculated by additional inter-sample nucleotide diversity calculations.

### Gene annotation (methyltransferase (MTase) and restriction enzyme (REase))

Gene predictions of prokaryotes, eukaryotes, and viruses of SHIPPO MAG were processed with GeneMarkS-2 (v. 1.09_1.07_lic) [80], GeneMark-ES [81], and Prodigal [82], respectively. We annotated the protein-coding sequences using GhostKOALA [83] to generate Kyoto Encyclopedia of Genes and Genomes orthology (KO) classification.

RMS pipeline identified the restriction-modification (RM) systems (https://github.com/oliveira-lab/RMS) using Hidden Markov model (HMM) profiles built from curated reference protein sequences by a type of RM system. In addition, genes related to the RM system were obtained by its type through KO terms, as follows:

- Type I RM system (K01153; type I REase, R subunit [EC:3.1.21.3], K01154; type I REase, S subunit [EC:3.1.21.3], K03427; type I REase M protein [EC:2.1.1.72])
- Type II RM system (K01155; type II REase [EC:3.1.21.4], K06223; DNA adenine methylase [EC:2.1.1.72], K13581; modification methylase [EC:2.1.1.72], K07317; adenine-specific DNA-MTase [EC:2.1.1.72], K07318; adenine-specific DNA-MTase [EC:2.1.1.72], K07319; adenine-specific DNA-MTase[EC:2.1.1.72], K00571; site-specific DNA-MTase (adenine-specific) [EC:2.1.1.72], K00558; DNA (cytosine-5)-MTase 1 [EC:2.1.1.37], K00590; site-specific DNA-MTase (cytosine-N4-specific) [EC:2.1.1.113])
- Type III RM system (K01156; type III REase [EC:3.1.21.5], K07316; adenine-specific DNA-MTase [EC:2.1.1.72])
- Type IV RM system (K07451; 5-methylcytosine-specific REase A [EC:3.1.21.-], K07452; 5-methylcytosine-specific REase B [EC:3.1.21.-], K19147; 5-methylcytosine-specific REase subunit McrC, K07448; restriction system protein)

The RM system type was classified by searching genes encoding the MTase and REase that were < 10 genes apart.

### Host-prediction

We conducted multiple strategies for viral host prediction to identify potential microbial hosts of the vOTUs from SHIPPO MAG. (1) Sequence homology matches: SHIPPO vOTU genomes were aligned against SHIPPO PRO MAG using BLASTn. Among the best hits, hits that met the criterion of ≥ 75% identity with alignment length ≥2500 bp were considered. (2) Clustered Regularly Interspaced Short Palindromic Repeats (CRISPR) spacer matches: CRISPR spacers were identified using CRT (CLI v. 1.2) [84] and PILER-CR (v. 1.06) [85] using the criteria established in the Microbial Genome Annotation Pipeline (MiGAP v. 4) [86]. Spacer sequences were compared by all-vs.-all BLASTn with “-task BLASTn-short -evalue 1e-10-perc_identity 95” parameters. (3) tRNA matches: transporter RNAs were obtained using ARAGORN (v. 1.2.38) [87] and compared by all-vs.-all BLASTn with “-perc_identity 100” parameter. Only BLAST hits were considered with 100% coverage query length of the alignment. We matched the host of SHIPPO vOTU against SHIPPO PRO MAG through the filtered results from the three abovementioned strategies.

### DNA modification and motif detection

DNA methylation was detected with a series of pipelines from BaseMod (https://github.com/ben-lerch/BaseMod-3.0). Briefly, each long-read of samples was mapped to the SHIPPO MAG sequences using PBAlign. Then, the modified positions of genomes were identified through ipdSummary (v. 2.0), which is a *t* test based comparison of interpulse duration ratios (IPDs) to the “synthetic control” model. These approaches used the same parameters documented in BaseMode. To find novel motifs methylated from each SHIPPO MAG, we found methylated sequence motifs by a de novo approach using MultiMotifMaker [14]. Additionally, we collected the previously curated recognition motifs of MTase from the Restriction Enzyme Database (REBASE; http://rebase.neb.com/cgi-bin/msublist) [13] to further capture methylated motifs in SHIPPO MAG.

### Meta-epigenomic analysis

Due to the specificity of the metagenome, insufficient reads were sequenced to calculate methylation for all SHIPPO MAGs. Therefore, using a relatively weak criterion of 10× coverage per strand and ≥30 modification quality value, we identified the overall *N*^6^-methyladenosine (m^6^A) and *N*^4^-methylcytosine (m^4^C) DNA modification trends throughout SHIPPO MAGs across the kingdoms of life. To complement this point, the ratio of methylated motifs for each genome in each sample was calculated only within a genomic breadth of coverage in 10×. Additionally, when the genome breadth of coverage was less than a certain percentage of each MAG size (SHIPPO PRO < 20%, SHIPPO EUK < 10%, SHIPPO vOTU < 60%, SHIPPO GV < 20%; according to genome size), they were excluded from further analysis.

MTase sequences were downloaded from REBASE (http://rebase.neb.com/cgi-bin/msubprolist), and the most similar MTase was searched for by querying the candidate MTase of SHIPPO MAG with a ≥50% identity parameter using BLASTp in REBASE. Afterward, we compared the motifs obtained from the methylation analysis of SMRT (which were > 20% methylated) sequencing with the recognition motif of the most similar reference MTase.

### Genome-wide epigenetic analysis

To compare DNA methylation profiles at single-nucleotide resolution across samples, we selected the most dominant MAGs (SHIPPO_PRO_33) that mapped ≥50% of the genomic breadth of 20× per strand in all samples. MAGs consist of fractionated genomes, making it difficult to understand the overall genomic structure, so we rearranged the order of the contigs through genome comparison with the closest reference genome using the Mauve Contig Mover [88]. Then, the replication origin (*ori*) and terminus (*ter*) were identified using the gc_skew.py script (https://github.com/christophertbrown/iRep) through the concatenate reordered contigs. In addition, methylation fractions (cellular fractions of DNA methylation) in regions overlapped by 20× in all samples were compared at the single nucleotide level.

### Motif enrichment

Motif enrichment analysis was processed by calculating the motif densities of three regions: regulatory, genic, and intergenic. Five hundred simulations were performed by shuffling the coding region based on the average gene length and the number of genes present in each genome strand, and the Shapiro test (*P* value > 0.05) was performed for each of the three regions. Next, *z*-scores were calculated for the motifs following normality and converted into *p*-values. Moreover, we used a multiscale signal representation (MSR) [89] method to assess whether the motif for specific regions is enriched or depleted (“section_2” of https://github.com/oliveira-lab/BEAST).

### Principal coordinate analysis (PCoA)

PCoA was performed based on the SNVs, methylated motifs, and methylation fractions across samples with the Bray–Curtis distance with R package “vegan” and visualized with “ggplot2”.

## Supplementary Information

**Additional file 1: Supplementary Figure S1**. SHIPPO samples in the northwest Pacific Ocean. **Supplementary Figure S2**. Compositional profile of microbial communities spanning eukaryotes, prokaryotes, and viruses. **Supplementary Figure S3**. The *Cand*. P. Giovannoni NP1 genome-wide distribution of methylated fractions. **Supplementary Figure S4**. The *Pelagibacter* (SHIPPO PRO 33) genome-wide distribution of methylated fractions. **Supplementary Figure S5**. GANTC motif density in the SHIPPO_PRO_33 genome. **Supplementary Figure S6**. Phylogenetic tree of eukaryotic plankton MAGs obtained from SHIPPO. **Supplementary Figure S7**. The GANTC methylation proportion comparison according to the genome fraction in six species belonging to the Alphaproteobacteria. **Supplementary Figure S8**. Benchmarking of viral taxonomy classification according to the number and fraction of viral genes. **Supplementary Table S1**. SHIPPO samples in the northwest Pacific Ocean. **Supplementary Table S2**. Methylated motifs of SHIPPO MAGs. **Supplementary Table S3**. Unmethylated GANTC sites across samples.

**Additional file 2: Supplementary Data 1**. Genome information of SHIPPO MAGs. **Supplementary Data 2**. (DOI link: https://doi.org/10.6084/m9.figshare.17161715). Genome files of SHIPPO MAGs.

**Additional file 3: Supplementary Data 3**. RM system of MAGs from ocean metagenomic studies.

**Additional file 4: Supplementary Data 4**. Methylation profiles of 5-mer motifs across MAGs and sampling stations.

## Data Availability

The raw Illumina and SMRT sequence files used in this study were deposited in the NCBI BioProject under the accession PRJNA784005. Genome files of SHIPPO MAGs are provided online at Figshare (Supplementary Data 2; 10.6084/m9.figshare.17161715). Analysis codes of the meta-epigenomics are available at the repository (https://github.com/hoonjeseong/Meta-epigenomics) under a MIT license.

## Abbreviations

MTase: Methyltransferase
RM: Restriction–modification
REase: Restriction enzyme
SMRT: Single-molecule real-time
m^6^A: *N*^6^-methyladenosine
m^5^C: *N*^4^-methylcytosine
SHIPPO: Shipborne Pole-to-Pole Observations
PacBio: Pacific Biosciences
MAG: Metagenome-assembled genome
MIMAG: Minimum Information about a Metagenome-assembled Genome
GORG-Tropics: Global Ocean Reference Genomes Tropics
GTDB: Genome Taxonomy Database
REBASE: Restriction Enzyme database
*F*ST: Fixation index
IPD: Interpulse duration
SNMV: Single nucleotide methylation variation
SNV: Single nucleotide variant
*ori*: Replication origin
*ter*: Replication terminus
vOTUs: Viral operational taxonomic units
PCoA: Principal coordinate analysis
