# Supplementary material for: Marine DNA methylation patterns are associated with microbial community composition and inform virus-host dynamics
Source: Microbiome. 2022 Sep 28;10:157. doi: 10.1186/s40168-022-01340-w (PMC9516812; doi:10.1186/s40168-022-01340-w)
Supplement: Supplementary file 2 — Additional file 1: Supplementary Figure S1. SHIPPO samples in the northwest Pacific Ocean. Supplementary Figure S2. Compositional profile of microbial communities spanning eukaryotes, prokaryotes, and viruses. Supplementary Figure S3. The Cand. P. Giovannoni NP1 genome-wide distribution of methylated fractions. Supplementary Figure S4. The Pelagibacter (SHIPPO PRO 33) genome-wide distribution of methylated fractions. Supplementary Figure S5. GANTC motif density in the SHIPPO_PRO_33 genome. Supplementary Figure S6. Phylogenetic tree of eukaryotic plankton MAGs obtained from SHIPPO. Supplementary Figure S7. The GANTC methylation proportion comparison according to the genome fraction in six species belonging to the Alphaproteobacteria. Supplementary Figure S8. Benchmarking of viral taxonomy classification according to the number and fraction of viral genes. Supplementary Table S1. SHIPPO samples in the northwest Pacific Ocean. Supplementary Table S2. Methylated motifs of SHIPPO MAGs. Supplementary Table S3. Unmethylated GANTC sites across samples. [file 40168_2022_1340_MOESM1_ESM.docx]

**Additional File 1**

**Supplementary Information**

**Community-wide epigenetics provides novel perspectives on the ecology and evolution of marine microbiome**

Hoon Je Seong^1^, Simon Roux^2^, Chung Yeon Hwang^3^, Woo Jun Sul^1^*

Correspondence to: sulwj[@cau.ac.kr](mailto:xxxxx@xxxx.xxx)

**This file contains:**

1. **Supplementary Text:** Additional text is included regarding several analyses.
2. **Supplementary Figures:** A total of 8 supplementary figures are included here with legends.
3. **Supplementary Tables:** A total of 3 supplementary tables are included here with legends.
4. **Supplementary Data legends:** A total of 4 supplementary data legends are included here.

**Supplementary Text**

1. **DNA methylome measurement in metagenomic data**

The existing methylated motif (methylome) analysis focused on the isolated bacterial genome, which allowed to determine the correct methylated motif from the sufficient coverage of SMRT sequencing. However, because metagenomic data is widely distributed in the sequences of various organisms, reads could not cover the entire genome with sufficient coverage, which is a limitation in analyzing the methylome using existing methods. Therefore, we considered setting the methylome analysis in the metagenomic study to minimize the error of methylome measurement by changing the mapped coverage threshold.

We used the genomes of six bacteria belonging to the Alphaproteobacteria obtained from the Blow *et al*. study*^20^* to benchmark the DNA methylome measurement. Based on the methylated GANTC proportion (the number of a methylated motif/the number of a motif) of the entire genome, we compared how much the methylated GANTC proportion changes depending on the fraction on the genome. The methylation proportion was calculated on the region of the genome fraction selected randomly according to the threshold 1~95% with 100 iterations each.

We calculated the residuals between the predicted value (methylation proportion by genome fraction) and the measured value (methylation proportion of entire genome) (Supplementary Fig. 7). We found that calculating the methylation profile using fractions of 20% or more of the genome is a method with minimal error. These results demonstrate the potential to analyze the profile of methylation motifs in various organisms even with fragmented parts of the metagenomic data.

1. **Benchmarking of viral taxonomy classification**

Considering that it is difficult to classify the taxonomy of most marine viruses, we performed a family-level classification benchmarking test to obtain the optimal parameters to minimize false positives. Viral MAGs (vMAGs) constituted 5,282 vOTU clusters with genomes from previous viral genome datasets, of which only 1,176 vOTU clusters were classified at the family level through the GOV2.0 study*^66^* (Not a single Earth virome genome composed of vOTUs with vMAG has been classified). A total of 3,575 vMAGs belonging to 1,176 vOTU clusters were considered true positives (*TP*) when the Demovir results were consistent. Conversely, if the result of vMAG classified through Demovir was an incorrect assignment compared to the family classification of GOV2.0, it was considered a false positive (*FP*).

Precision quantified the quality through the Demovir classification approach in terms of the reliability of its assignment. In addition, the prevalence value was calculated to determine how many vMAGs could be classified appropriately (*TP*) according to the threshold of the number and fraction of viral genes in each vMAG.

$$\mathrm{Precision}=\frac{TP}{TP+FP} \mathrm{Prevalence}= \frac{TP}{Total number of vMAGs}$$

We compared the precision and prevalence values to reduce *FP*s and classify as many genomes as possible. When 10% of the genes in each vMAG were viral, the precision value was 92.93, and 89.76% of the vMAGs were correctly classified. Moreover, when 15% of the genes in each vMAG were viral, the precision value was 94.31, and 85.45% of the vMAGs were correctly classified (Supplementary Fig. 8). We have demonstrated that the classification approach using the percentage of viral genes in the genome is efficient, and we believe that it will be helpful to classify unidentified viral genomes.


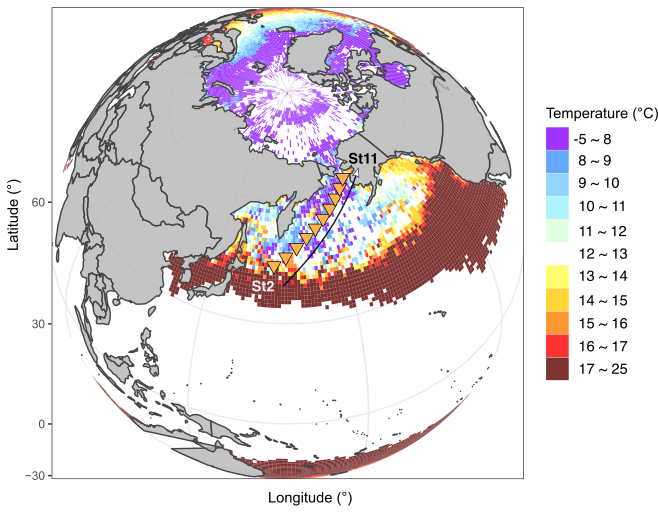


**Supplementary Fig. 1. SHIPPO samples in the northwest Pacific Ocean.**

The yellow triangle represents the 10 sampling stations (Station2–Station11: St2–St11) visited by the icebreaker *ARAON*, which sailed to the North Pole in 2015. The sea surface temperature records for July 2015 were downloaded from NEO and visualized (<https://neo.gsfc.nasa.gov/view.php?datasetId=MYD28M>). SHIPPO: Shipborne Pole-to-Pole Observations; NEO: NASA Earth Observations.


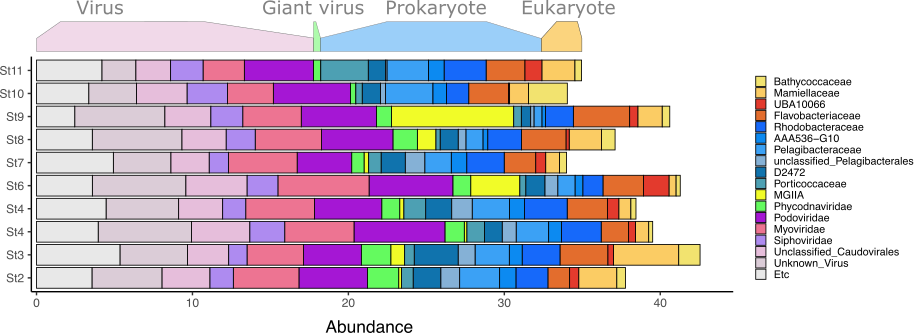


**Supplementary Fig. 2. Compositional profile of microbial communities spanning eukaryotes, prokaryotes, and viruses.**

Each colored bar represents the read-mapped percentage to the phylum level of the assembled genome; the *y*-axis indicates sampling stations St2–St11.


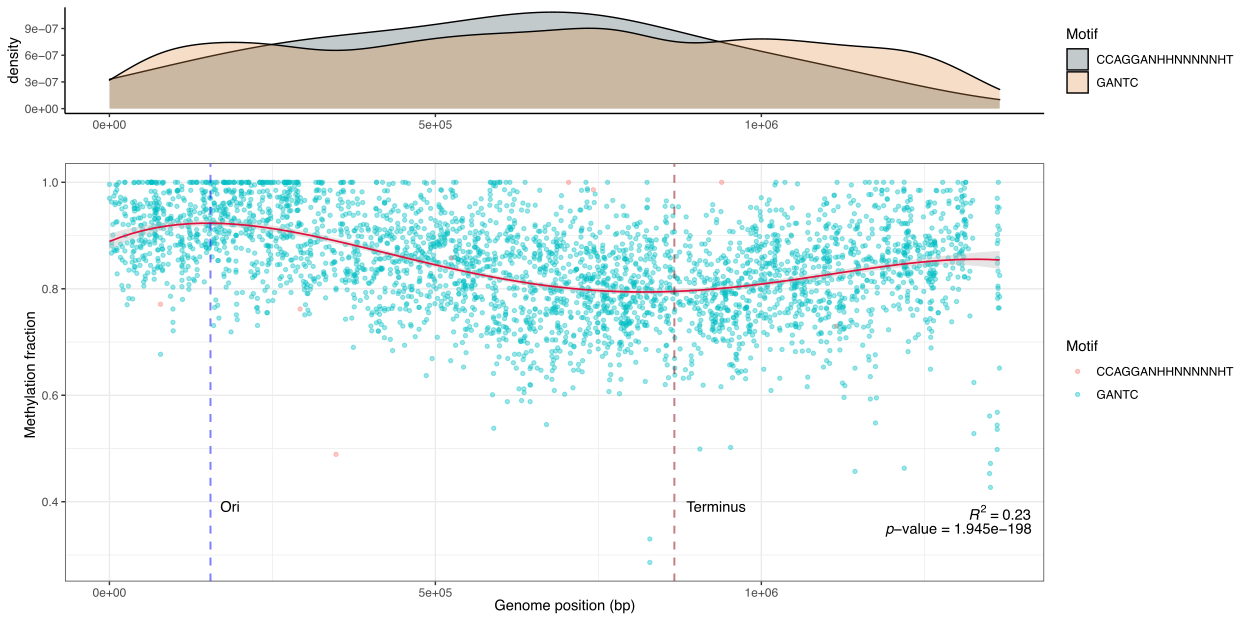


**Supplementary Fig. 3. The *Cand*. P. Giovannoni NP1 genome-wide distribution of methylated fractions.**

The genome-wide distribution of methylated fractions for GANTC and CCAGGAHHNNNNNHT motifs throughout the *Candidatus* Pelagibacter Giovannoni NP1 genome.


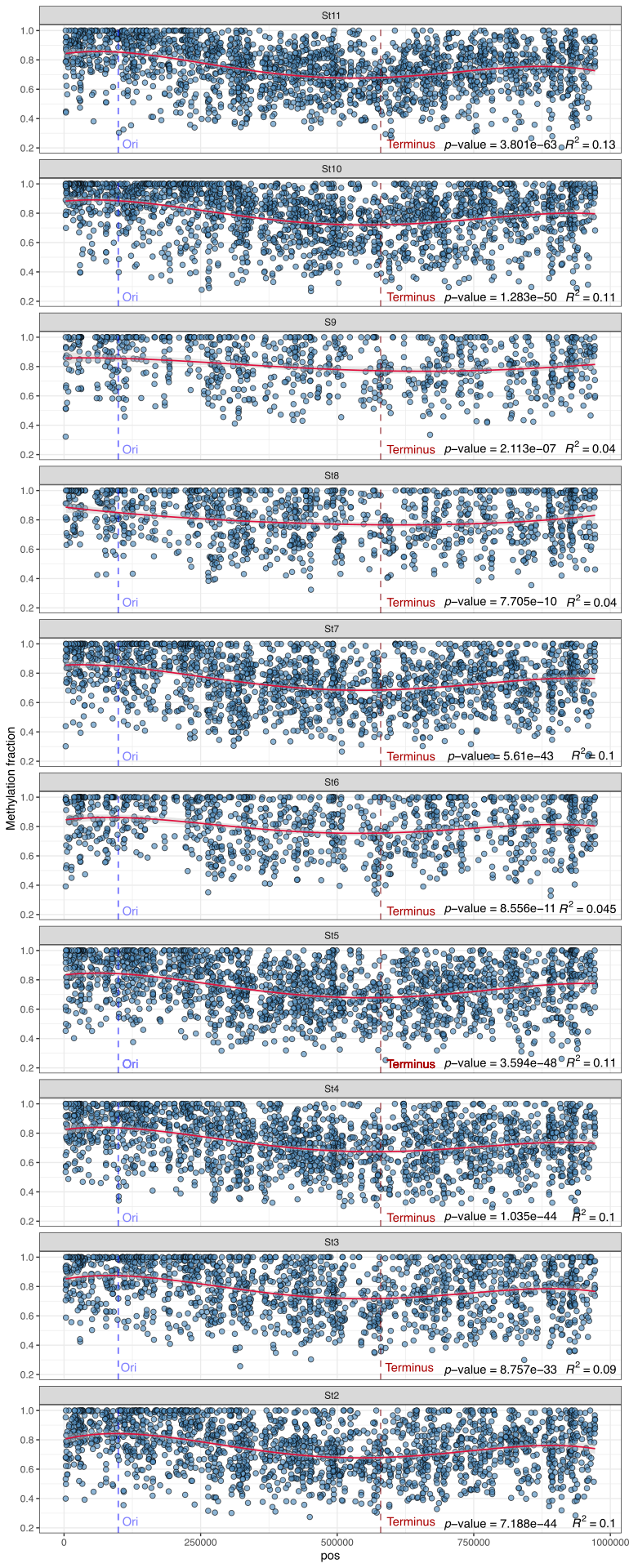
 **Supplementary Fig. 4. The *Pelagibacter* (SHIPPO PRO 33) genome-wide distribution of methylated fractions.**

The genome-wide distribution of methylated fractions for GANTC motifs throughout the SHIPPO PRO 33 genome across stations. SHIPPO: Shipborne Pole-to-Pole Observations.


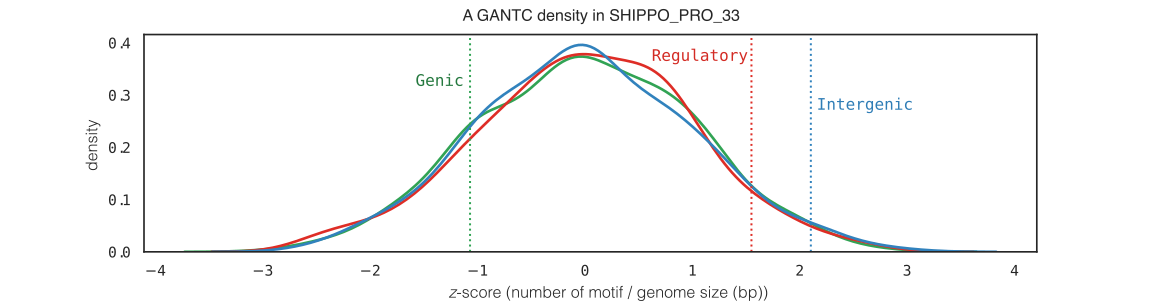


**Supplementary Fig. 5. GANTC motif density in the SHIPPO_PRO_33 genome.**

A randomized density of GANTC motif on the genic, intergenic, and regulatory regions throughout the *Pelagibacter* metagenome-assembled genome (SHIPPO_PRO_33) were calculated as *z*-scores. The dotted lines represent the *z*-scores for the empirical density of GANTC motifs on these regions. SHIPPO: Shipborne Pole-to-Pole Observations.


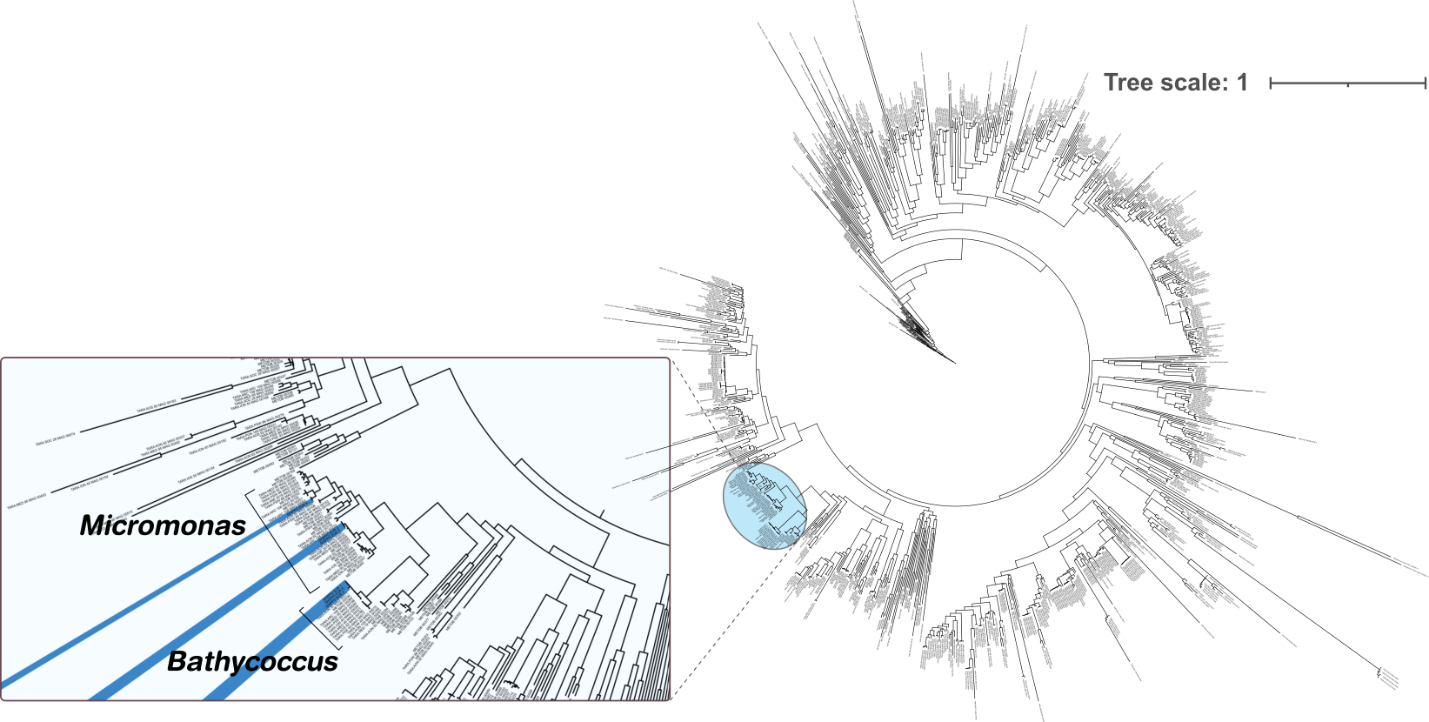


**Supplementary Fig. 6. Phylogenetic tree of eukaryotic plankton MAGs obtained from SHIPPO.**

The phylogenetic trees of MAGs were reconstructed by RNA polymerase protein sequences. Taxonomy of a total of six strain-level eukaryotic MAGs (colored blue) was inferred by comparing RNA polymerase from Delmont *et al.^15^*. MAG: metagenome-assembled genome; SHIPPO: Shipborne Pole-to-Pole Observations.


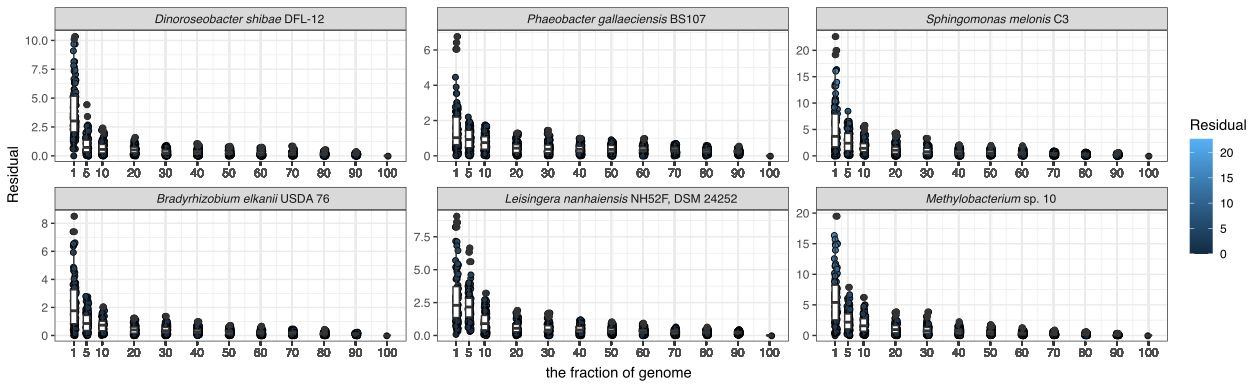


**Supplementary Fig. 7. The GANTC methylation proportion comparison according to the genome fraction in six species belonging to the Alphaproteobacteria.**


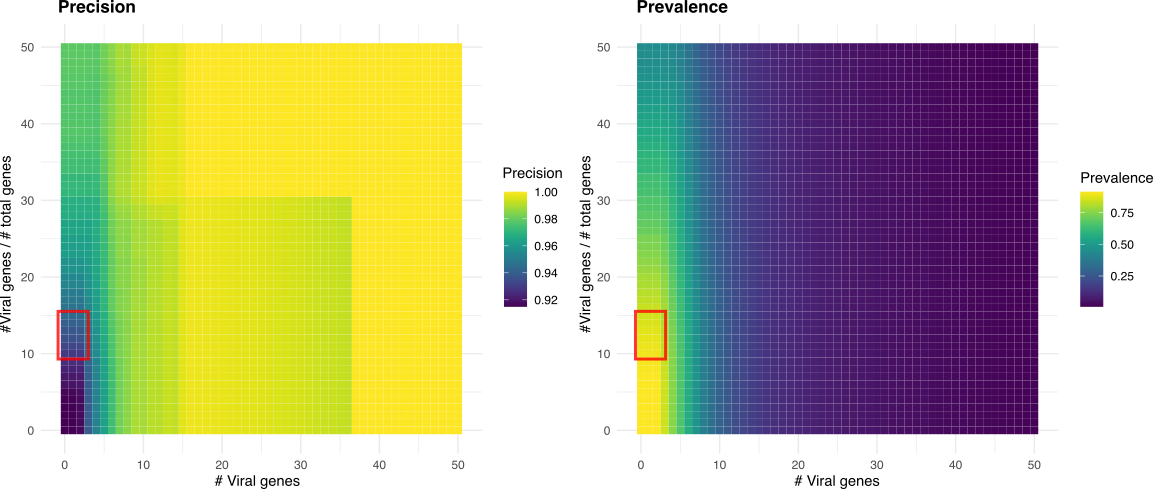


**Supplementary Fig. 8. Benchmarking of viral taxonomy classification according to the number and fraction of viral genes.**

**Supplementary Table 1. SHIPPO samples in the northwest Pacific Ocean.**

| Station | Date | Latitude (°) | Longitude (°) | Depth (m) | Temperature (°C) | Salinity (psu) |
| --- | --- | --- | --- | --- | --- | --- |
| St2 | 2015-07-21 | 44.38 (N) | 155.35 (E) | 7 | 12.09 | 32.15 |
| St3 | 2015-07-22 | 46.60 (N) | 159.43 (E) | 7 | 9.46 | 32.23 |
| St4 | 2015-07-23 | 48.86 (N) | 163.52 (E) | 7 | 10.09 | 32.27 |
| St5 | 2015-07-24 | 51.10 (N) | 167.60 (E) | 7 | 10.71 | 32.33 |
| St6 | 2015-07-24 | 53.08 (N) | 171.64 (E) | 7 | 11.47 | 32.17 |
| St7 | 2015-07-25 | 55.07 (N) | 175.68 (E) | 7 | 12.20 | 32.34 |
| St8 | 2015-07-26 | 57.05 (N) | 179.73 (E) | 7 | 12.26 | 32.17 |
| St9 | 2015-07-27 | 59.03 (N) | 176.23 (W) | 7 | 12.55 | 32.16 |
| St10 | 2015-07-28 | 61.02 (N) | 172.19 (W) | 7 | 11.05 | 30.54 |
| St11 | 2015-07-29 | 63.00 (N) | 168.15 (W) | 7 | 10.50 | 31.18 |

SHIPPO: Shipborne Pole-to-Pole Observations.

**Supplementary Table 2. Methylated motifs of SHIPPO MAGs.**

| **MAGs** | **Family** | **Methylated motifs (forward/reverse)** | **REBASE motif** | **MTase Type** |
| --- | --- | --- | --- | --- |
| SHIPPO_PRO_101 | MGIIA | GGTAG/None, CGCG/CGCG, TCGCGA/TCGCGA, GGTAG/CTACC | CGCG | Type II MTase |
| SHIPPO_PRO_102 | MGIIA | CGCG/CGCG | CGCG | Type II MTase |
| SHIPPO_PRO_132 | *Microbacteriaceae* | TTAA/TTAA, ATTAAT/ATTAAT, GTTAAT/None | TTAA | Type II MTase |
| SHIPPO_PRO_22 | *Rhodobacteraceae* | GANTC/GANTC, GASTC/GASTC, GAWTC/GAWTC, GAGTC/None | GANTC | Type II MTase |
| SHIPPO_PRO_241 | AAA536-G10 | GANTC/GANTC, GASTC/GASTC, GAWTC/GAWTC, GAGTC/None | GANTC | Type II MTase |
| SHIPPO_PRO_245 | *Pelagibacteraceae* | GANTC/GANTC, GASTC/GASTC, GAWTC/GAWTC, GAGTC/None, CGAN_6_AATC/GATTN_6_TCG, CGAYN_4_TTCA/TGAAN_4_RTCG, GAAN_6_TCGG/CCGAN_6_TTC, CGAN_6_GTCY/RGACN_6_TCG, GAAN_5_TCGA/TCGAN_5_TTC | GANTC | Type II MTase |
| SHIPPO_PRO_246 | *Pelagibacteraceae* | GANTC/GANTC, GASTC/GASTC, GAWTC/GAWTC, GAGTC/None, CGAAN_5_TCC/GGAN_5_TTCG | GANTC | Type II MTase |
| SHIPPO_PRO_33 | *Pelagibacteraceae* | GANTC/GANTC, GASTC/GASTC, GAWTC/GAWTC, GAGTC/None, CGAN_7_CTTG/CAAGN_7_TCG, TGACN_5_CTC/GAGN_5_GTCA, GTGAN_5_RTCG/CGAYN_5_TCAC, CCGCAG/None, ATGN_5_GTCG/CGACN_5_CAT, CGAN_6_GTCY/RGACN_6_TCG | GANTC | Type II MTase |
| SHIPPO_PRO_64 | o_*Pelagibacteraceae* | GANTC/GANTC, GASTC/GASTC, GAWTC/GAWTC, GAGTC/None, GAGN_6_TAYG/CRTAN_6_CTC | GANTC | Type II MTase |
| SHIPPO_PRO_66 | AAA536-G10 | GANTC/GANTC, GASTC/GASTC, GAWTC/GAWTC, GAGTC/None | GANTC | Type II MTase |
| vOTU_N_648 | Unknown_Virus | GATC/GATC, GGATC/None, None/GATCC, TGATCA/TGATCA, RGATCY/RGATCY, GGATC/GATCC | GATC | Type II MTase |
| vOTU_sg_10720 | *Siphoviridae* | GGAG/None, GRRGA/None, GGWGA/None, GAAGATCTTC | CTYCAG | Type II MTase |

SHIPPO: Shipborne Pole-to-Pole Observations; MAG: metagenome-assembled genome; MTase: methyltransferase.; underscore: DNA modification

**Supplementary Table 3. Unmethylated GANTC sites across samples.**

| **Position (Contig:Position:Strand)** | **Region** | **KEGG** |
| --- | --- | --- |
| SHIPPO_PRO_33_11:21967:- | Genic | K00215 |
| SHIPPO_PRO_33_10:5600:- | Genic | K00285 |
| SHIPPO_PRO_33_1:33961:+ | Genic | K00666 |
| SHIPPO_PRO_33_1:34117:+ | Genic | K00666 |
| SHIPPO_PRO_33_4:47162:+ | Genic | K01879 |
| SHIPPO_PRO_33_10:61000:+ | Genic | K01956 |
| SHIPPO_PRO_33_10:61092:+ | Genic | K01956 |
| SHIPPO_PRO_33_5:70038:- | Genic | K03702 |
| SHIPPO_PRO_33_11:32121:+ | Genic | K03980 |
| SHIPPO_PRO_33_6:5815:+ | Genic | K07090 |
| SHIPPO_PRO_33_1:85989:+ | Genic | K14980 |
| SHIPPO_PRO_33_2:49451:+ | Genic | Unclassified |
| SHIPPO_PRO_33_10:26785:- | Genic | Unclassified |
| SHIPPO_PRO_33_11:155821:+ | Genic | Unclassified |
| SHIPPO_PRO_33_1:88695:- | Intergenic | Unclassified |
| SHIPPO_PRO_33_1:33963:- | Intergenic | Unclassified |
| SHIPPO_PRO_33_1:34119:- | Intergenic | Unclassified |
| SHIPPO_PRO_33_1:85991:- | Intergenic | Unclassified |
| SHIPPO_PRO_33_2:49453:- | Intergenic | Unclassified |
| SHIPPO_PRO_33_2:7767:- | Intergenic | Unclassified |
| SHIPPO_PRO_33_3:78935:+ | Intergenic | Unclassified |
| SHIPPO_PRO_33_3:57851:+ | Intergenic | Unclassified |
| SHIPPO_PRO_33_3:4705:- | Intergenic | Unclassified |
| SHIPPO_PRO_33_5:70036:+ | Intergenic | Unclassified |
| SHIPPO_PRO_33_6:5817:- | Intergenic | Unclassified |
| SHIPPO_PRO_33_8:61449:+ | Intergenic | Unclassified |
| SHIPPO_PRO_33_10:26783:+ | Intergenic | Unclassified |
| SHIPPO_PRO_33_10:5598:+ | Intergenic | Unclassified |
| SHIPPO_PRO_33_10:61094:- | Intergenic | Unclassified |
| SHIPPO_PRO_33_11:171226:+ | Intergenic | Unclassified |
| SHIPPO_PRO_33_11:146653:+ | Intergenic | Unclassified |
| SHIPPO_PRO_33_11:21965:+ | Intergenic | Unclassified |
| SHIPPO_PRO_33_11:86684:- | Intergenic | Unclassified |
| SHIPPO_PRO_33_11:99001:- | Intergenic | Unclassified |
| SHIPPO_PRO_33_11:32123:- | Intergenic | Unclassified |
| SHIPPO_PRO_33_3:78937:- | Regulatory | K02864 |
| SHIPPO_PRO_33_5:1650:- | Regulatory | K04077 |
| SHIPPO_PRO_33_6:11812:- | Regulatory | K04488 |
| SHIPPO_PRO_33_8:6914:- | Regulatory | K07462 |
| SHIPPO_PRO_33_11:114352:- | Regulatory | K09883 |
| SHIPPO_PRO_33_8:61451:- | Regulatory | K09969 |
| SHIPPO_PRO_33_5:61241:- | Regulatory | Unclassified |

SHIPPO: Shipborne Pole-to-Pole Observations; KEGG: Kyoto Encyclopedia of Genes and Genomes.

**Supplementary Data legends**

**Additional File 2: Supplementary Data 1** (Microsoft Excel format). Genome information of SHIPPO MAGs.

**Supplementary Data 2** (DOI link: https://doi.org/10.6084/m9.figshare.17161715). Genome files of SHIPPO MAGs.

**Additional File 3: Supplementary Data 3** (Microsoft Excel format). RM system of MAGs from ocean metagenomic studies.

**Additional File 4: Supplementary Data 4** (Microsoft Excel format). Methylation profiles of 5-mer motifs across MAGs and sampling stations.
